# Ultra-antireflective synthetic brochosomes

**DOI:** 10.1038/s41467-017-01404-8

**Published:** 2017-11-03

**Authors:** Shikuan Yang, Nan Sun, Birgitt Boschitsch Stogin, Jing Wang, Yu Huang, Tak-Sing Wong

**Affiliations:** 1 Department of Mechanical and Nuclear Engineering, The Pennsylvania State University, University Park, PA 16802 USA; 2 Materials Research Institute, The Pennsylvania State University, University Park, PA 16802 USA; 3 Institute for Composites Science Innovation, School of Materials Science and Engineering, Zhejiang University, Hangzhou, 310027 China; 4 Department of Biomedical Engineering, The Pennsylvania State University, University Park, PA 16802 USA

## Abstract

Since the early discovery of the antireflection properties of insect compound eyes, new examples of natural antireflective coatings have been rare. Here, we report the fabrication and optical characterization of a biologically inspired antireflective surface that emulates the intricate surface architectures of leafhopper-produced brochosomes—soccer ball-like microscale granules with nanoscale indentations. Our method utilizes double-layer colloidal crystal templates in conjunction with site-specific electrochemical growth to create these structures, and is compatible with various materials including metals, metal oxides, and conductive polymers. These brochosome coatings (BCs) can be designed to exhibit strong omnidirectional antireflective performance of wavelengths from 250 to 2000 nm, comparable to the state-of-the-art antireflective coatings. Our results provide evidence for the use of brochosomes as a camouflage coating against predators of leafhoppers or their eggs. The discovery of the antireflective function of BCs may find applications in solar energy harvesting, imaging, and sensing devices.

## Abstract (web summary)

New examples of natural antireflective coatings are rare. Here, Yang et al. report the fabrication and optical characterization of a biologically inspired antireflective surface that emulates the surface architecture of the leafhopper-produced brochosomes with antireflective performance from 250 to 2000 nm.

## Introduction

**Fig. 1:**
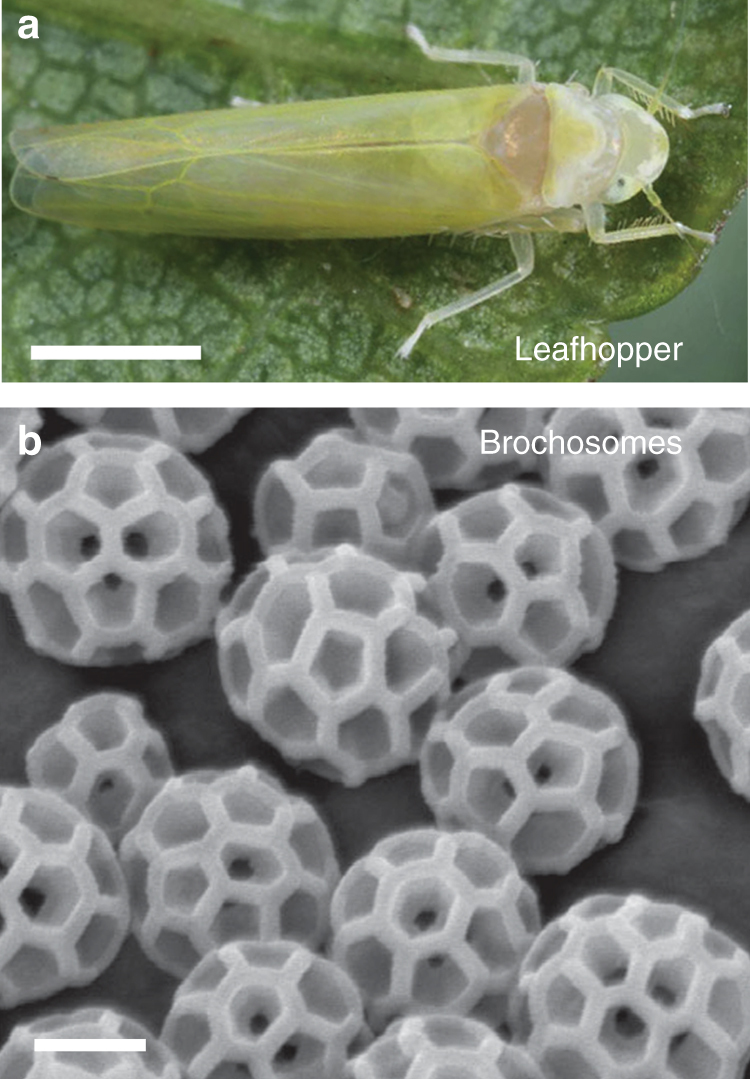
Leafhopper and its brochosomes. **a** Optical image of a leafhopper, *Alnetoidia alneti* (Dahlbom), and **b** an electron micrograph showing its brochosomes. Both images are reprinted from ref. ^19^, by permission of the Royal Society. Scale bars, 1 mm (leafhopper) and 200 nm (brochosomes)

Natural surfaces have demonstrated how different micro/nanoscale surface architectures can yield an array of distinct interfacial functions^1–9^. While many of these surface structures can now be manufactured using advanced manufacturing techniques^10–13^, scalable fabrication methods capable of producing a number of these natural structures have remained elusive. Among these natural structures are leafhopper-produced brochosomes (Fig. 1)^14–16^. Naturally occurring integumental brochosomes are microscale granules with nanoscale surface indentations arranged in a honeycomb pattern, making the geometry of a brochosome particle similar to those of a soccer ball^17^. Leafhoppers living in different regions create brochosomes with significantly varied structural geometries, with distinct diameters and numbers of pits^18^. In addition to their use as non-sticking coatings^19,20^, the intricate nanoscale architecture and three-dimensional periodicity of these BCs suggest they may have complex optical properties. Interestingly, Swain proposed in 1936 that these brochosomes might serve as a camouflage coating to hide the eggs of leafhoppers from their predators or parasites^21^, but no experimental evidence has been shown thus far. This is due to the fact that the optical functions of the BCs remained minimally understood as large quantities of brochosomes for systematic study are not readily producible. Until now, micro/nanomanufacturing techniques to create brochosomes of various geometries and material compositions have not been available.

Here, we develop a fabrication concept for preparing BCs composed of closely packed artificial brochosomes, whose shape and geometry closely mimic the natural ones created by leafhoppers. We utilize double-layer colloidal crystal (DCC) templates combined with site-specific electrochemical growth to prepare artificial BCs comprising metals, metal oxides, polymers, or their hybrids on any conductive substrates. The structure of the synthetic BCs, defined by the diameter of the brochosomes, the inter-brochosome distance, as well as the size and depth of the pits within the brochosomes, can be precisely engineered, allowing us to systematically explore their structure–property relationships. Using silver (Ag) as a model material, we have shown that 2-µm thick Ag BCs (i.e., BCs comprise 2 µm diameter brochosomes) are capable of reflecting <~1% on average of any wavelength in the 250–2000 nm optical window. This reflectance is comparable to those of the state-of-the-art synthetic antireflective materials^22^. The superior antireflection is attributed to the unique structural geometries of the brochosomes, as demonstrated experimentally and numerically. Additionally, our experimental results suggest a possible use of BCs as a camouflage and protective layer for leafhoppers or their eggs against potential predators in their natural habitats^18,23,24^.

## Results

### Fabrication of synthetic brochosomal coatings

**Fig. 2:**
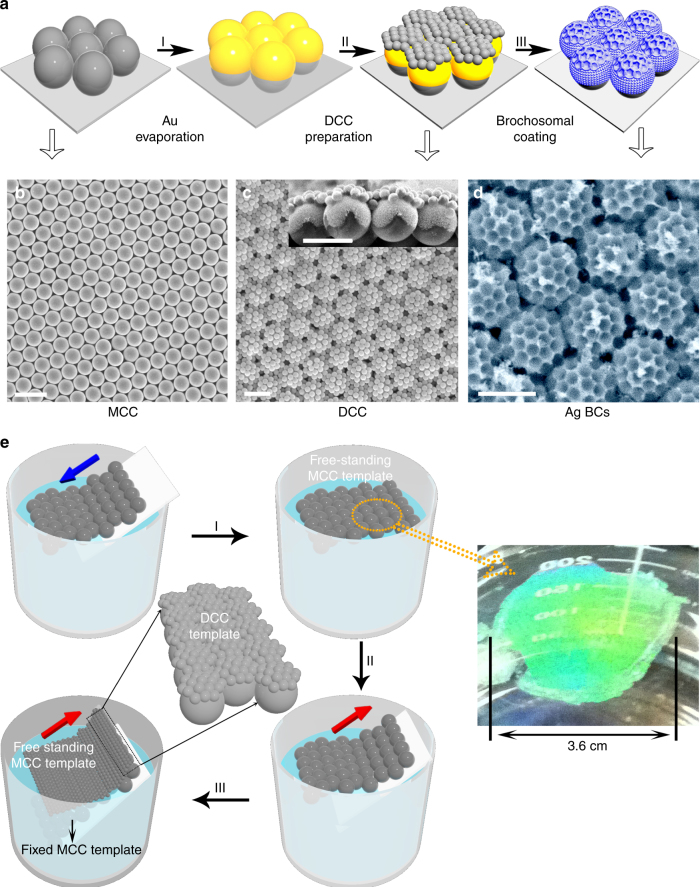
Fabrication of synthetic brochosomal coating. **a** Fabrication process of the synthetic BCs. I. Gold evaporation. II. Preparation of DCC template. III. DCC template-based site-specific electrochemical deposition which creates BCs after PS spheres removal. **b**–**d** SEM images of MCC template (scale bar, 2 µm), DCC template (scale bar, 2 µm, and inset, 2 µm), and Ag BCs (scale bar, 2 µm), respectively. **e** Fabrication process of DCC template. I. MCC template transferred onto the air/water interface. II. MCC template transferred onto another arbitrary substrate. III. DCC template created by transferring another layer of MCC template onto a pre-fabricated MCC template that was thermally anchored on a substrate. Note that the thermal treatment of the MCC template around the glass transition temperature increases the interfacial area, thereby enhancing the binding between the PS spheres and the silicon substrate

Our synthetic BCs were created by first preparing a highly ordered DCC template, followed by site-specific electrodeposition on the template (Fig. 2a–d). The DCC template was prepared through a layer-by-layer stacking process (Fig. 2e). First, a monolayer colloidal crystal (MCC) template (>2 cm^2^) composed of polystyrene (PS) spheres was prepared by spin-coating, and was then transferred onto the water/air interface (Process I in Fig. 2e)^25–27^. An arbitrary substrate was used to pick up the free-standing MCC template from underneath (Process II in Fig. 2e, b). The substrate could be in the form of a smooth, flexible, curved, or roughened surface. A thin layer of Au film (~100 nm) was then deposited onto the MCC template (Process I in Fig. 2a). Next, another layer of MCC template comprising smaller PS spheres was transferred onto the Au-coated MCC template (Process III in Fig. 2e), yielding the DCC template (Process II in Fig. 2a, c), which was then used as the working electrode to perform electrochemical deposition of targeting materials. The electrodeposited material was constrained to grow conformally on the large PS spheres in the bottom layer template and continue to grow from the Au seed layer up and around the small PS spheres in the top layer template. After removing the PS spheres, BCs of the targeting materials can be obtained (Process III in Fig. 2a, d). The final structures closely mimic those of the natural brochosomes.

**Fig. 3:**
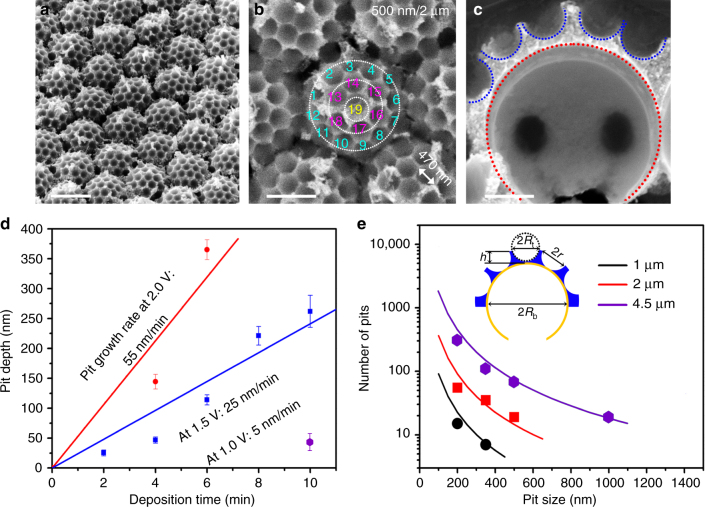
Structural control of BCs. **a**, **b** SEM images of Ag BCs at different magnifications. Scale bars: (**a**) 2 µm, and (**b**) 1 µm. **c** Cross-sectional image of a silver brochosome particle. Scale bar: 500 nm. **d** A plot showing the growth rate of pit depth at different electrodeposition voltages. Error bars represent standard deviations from 100 independent measurements of the pits. **e** A plot showing the relationship between the number of pits and the pit size on the brochosome particles for a given size of bottom layer spheres (labeled in inset). Solid lines indicate theoretical predictions. Inset showing the schematic of the relevant geometrical parameters of a brochosome-like particle

We used Ag as a model structural material to investigate the fabrication parameters of the synthetic BCs (Fig. 3). Adopting the DCC templating method, Ag BCs with a uniform structure can be prepared over a large area (>5 cm^2^). For example, synthetic Ag brochosomes with a diameter of ~2 µm were arranged in a hexagonally close-packed configuration (Fig. 3a). On this particular surface, there are ~19 pits hexagonally close-packed over the curved spherical surface (Fig. 3b), with the opening size and pit depth of 470 and 225 nm, respectively (Fig. 3c). This configuration is distinctively different from the conventional binary colloidal crystal templates^28^, which will typically result in ~12 pits located at the equators of the large spheres (Supplementary Fig. 1).

The availability of PS spheres of various sizes (from <100 nm to >10 µm) allows us to design synthetic BCs with a broad range of structural geometries (Supplementary Note 1). Using our technique, the pit number and size can be readily tailored by using PS spheres with different sizes to form the top layer MCC template (Supplementary Figs. 2, 3). The depth of the pits can be controlled by the electrodeposition time (Fig. 3d). Specifically, we found that the low growth rate at a low deposition voltage (e.g., 1.5 V) is preferred in order to accurately control the pit depth (Supplementary Figs. 4–7). In addition, we have demonstrated the formation of BCs using PS beads with diameters ranging from 1 to 4.5 µm with various pit sizes (Supplementary Figs. 8–10). Given the sizes of the PS spheres at the top and bottom layers of the DCC template, the geometrical parameters of the synthetic BCs can be precisely controlled. These geometrical parameters include the opening size (2*r*) and depth (*h*) of the pits, as well as the number of pits (*n*). We can predict *h* using the following equation (equation describing the height of a spherical cap): $h = R_{\mathrm{t}} - \left( {\sqrt {R_t^2 - r^2} } \right)$ if *h* < *R*_t_, where *R*_t_ is the radius of the top layer small PS spheres (Supplementary Fig. 11). In addition, the pit size (2*R*_t_), pit number (*n*), and the diameter (2*R*_b_) of the brochosomes can be related by $n \cong \frac{\pi }{{2\sqrt 3 }}\left( {\frac{{R_{\mathrm{b}}}}{{R_{\mathrm{t}}}}} \right)^2$, which agrees well with the experimental results (Fig. 3e).

### Optical characterizations

**Fig. 4:**
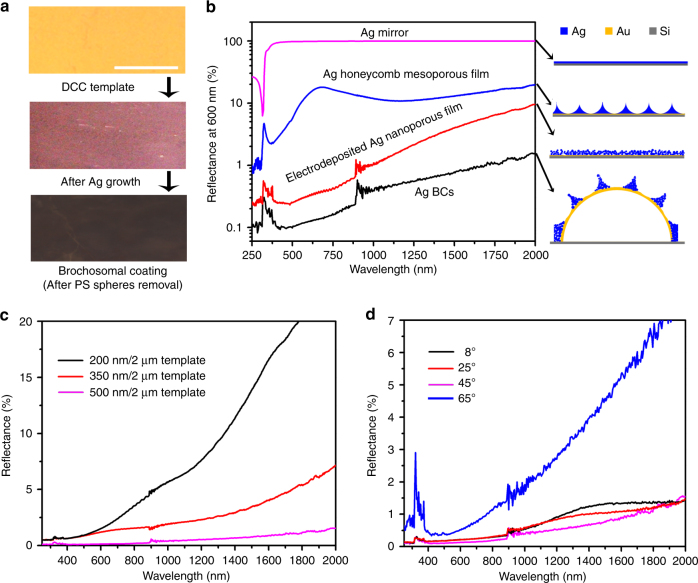
Antireflection performance of the synthetic BCs. **a** Optical images of DCC template before and after Ag electrochemical growth, and after the removal of PS beads. Scale bar: 5 mm. **b** A plot showing the reflection spectra of Ag mirror (in magenta), Ag honeycomb mesoporous film (in blue), Ag nanoporous film (in red) and Ag BCs (in black). Schematic drawings of the corresponding structures are shown on the right. The Ag BCs were prepared by using the DCC template composed of 2 µm large PS spheres with the top surface covered by 500 nm PS spheres. After electrodeposition, the pits of these BCs were 470 nm in opening size and 260 nm in depth. The Ag honeycomb mesoporous film was prepared by electrodeposition using 500 nm PS spheres on an Au-covered silicon wafer. The depth of the pits was ~250 nm (Supplementary Fig. 14). **c** A plot showing the reflection spectra of BCs with different pit sizes. **d** A plot showing the angle dependence of the reflection spectra of the Ag BCs composed of 2 µm particles with pits 470 nm in opening size and 260 nm in depth

Depending on the size of PS spheres in the top layer template, the as-deposited Ag BCs (with PS spheres) display different colors (Fig. 4a). The Ag BCs become dark in color after dissolving the PS spheres. The reflection spectra revealed that the reflection is continuously reduced as the pit depth is increased until *h* = 0.55*R*_t_ (Supplementary Fig. 12). Unlike planar metals which behave as excellent mirrors, our BCs consist of metal nanoparticle assemblies in the size range of 50–100 nm, which can further suppress reflection due to absorption by plasmon excitations^29,30^. The specular reflection on our BCs at an incident angle of 45° in the ultraviolet (UV) (250–380 nm), visible (Vis) (380–780 nm), and near infrared (NIR) (780–2000 nm) region is below 0.4%, 0.2%, and 1.4%, respectively, when the pit opening size is ~470 nm with a depth of ~260 nm, respectively (Fig. 4b). These values are substantially lower than those of the control samples, including a thermally evaporated planar Ag membrane, an Ag nanoporous film (Supplementary Fig. 13) prepared using the same electrodeposition method, and a planar Ag mesoporous film embedded with honeycomb arranged pits (Supplementary Fig. 14).

The antireflection of the 2 µm brochosome arrays can be further improved by modifying the pit size (Fig. 4c and Supplementary Note 2). In particular, optimized antireflection properties in the 250–2000 nm wavelength range can be achieved when the ratio of the pit depth and the diameter of the pit-creating PS spheres *h*/*R*_t_ is 0.6 according to FDTD simulation results^31^ (Supplementary Figs. 15, 16), which agrees well with the experimental results (i.e., *h*/*R*_t_ ~ 0.55) (Supplementary Fig. 12). The optimal pit depth to size ratio ensures efficient trapping of light by multiple internal reflections within the pit to suppress overall reflection of the coating^32,33^. In addition, our FDTD simulation results show that having a large number of pits (i.e., *n* > 7) on individual brochosomes is essential to further enhance the antireflection performance of the BCs (Supplementary Fig. 17), consistent with our experimental observations (i.e., *n* > 10), suggesting that more pits per unit projected surface area are desired to suppress reflection.

The antireflection property of the BCs is insensitive to the angle of the incident light (Fig. 4d and Supplementary Fig. 18). Specifically, as the incident angle varies from 8° to 45°, the reflectance from the BCs (*R*_b_ = 1 µm, *R*_t_ = 250 nm, *h* = 260 nm) is <0.25% in the visible region and <1.5% in the 700–2000 nm region. For an incident angle as large as 65°, the BCs still maintain low reflectance of <0.7% in the visible region, and <8% in the wavelength region of 700–2000 nm—a performance that is comparable to the state-of-the-art synthetic antireflective coatings^22^ (Supplementary Fig. 19). In comparison, the reflection spectrum of the planar mesoporous Ag film composed of hexagonally arranged nanovoids (equivalent to *R*_b_ → ∞, *R*_t_ = 250 nm, *h* = ~250 nm, see Supplementary Fig. 14) demonstrated angle dependence (Supplementary Fig. 20). This further shows that the radius of curvature (i.e., *R*_b_) of the individual synthetic brochosomes where the distributed pits are located is important for enhanced omnidirectional antireflection. Overall, our experimental measurements showed that strong omnidirectional antireflection (i.e., reflectance <10%) of BCs can be attained when ~9*λ* > *R*_b_> ~1.4*λ* (*λ* is wavelength of the electromagnetic waves), ~2*λ* > *R*_t_ > ~0.5*λ*, and *R*_t_/*R*_b_ < 0.35 (or *n* > ~7) (Supplementary Figs. 21, 22). Therefore, the pit geometry (*R*_t_, *h*) and number (*n*), as well as the brochosome size (*R*_b_) are important design parameters to achieve best omnidirectional antireflection of the BCs.

## Discussion

**Fig. 5:**
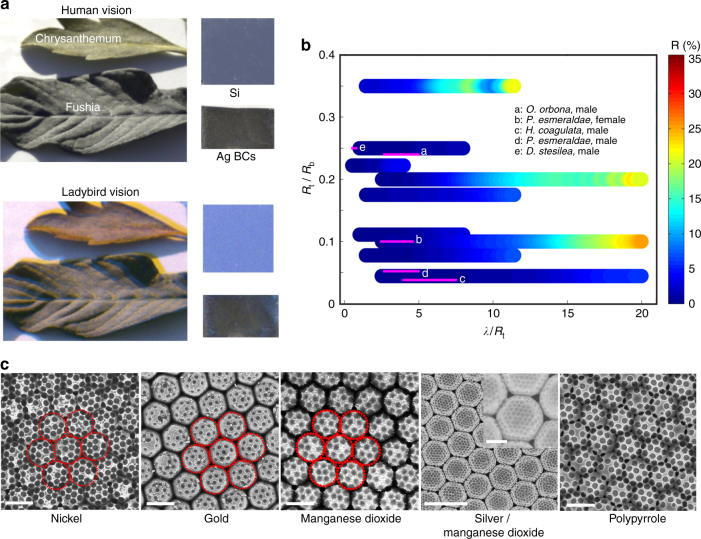
Possible camouflage function of BCs and their fabrication in different materials compositions. **a** Simulated human and ladybird beetle visions of BCs and two different leaf species. **b** Comparison of the structural parameters of natural and synthetic brochosomes for their optical reflectance; here, the color bar represents the experimental reflectance measurements of various synthetic BC structures and the magenta lines represent the natural brochosomes (i.e., the visible spectrum of the ladybird over *R*_t_ determines the line length and position along the *x*-axis). These natural brochosomes include a *Oncometopic orbona*, male. b *Proconia esmeraldae*, female. c *Homalodisca coagulata*, male. d *Proconia esmeraldae*, male. e *Diestostemma stesilea*, male (Supplementary Fig. 24). The observing (incident) angle is 45°. Note that the geometrical parameters of the aforementioned natural brochosomes are located at the low reflection region in the optical reflectance map obtained from the reflectance measurements of the synthetic brochosomes. The normalized plot allows one to identify the corresponding antireflection design parameters of the synthetic brochosome given a specific wavelength of light. **c** Scanning electron micrographs showing various BCs fabricated from nickel (Ni), gold (Au), manganese oxide (MnO_2_), silver/manganese oxide (Ag/MnO_2_), and polypyrrole (PPy). Scale bars: 2 µm (Ni, Au, MnO_2_, PPy) and 5 µm (Ag/MnO_2_, inset, 2 µm)

The ultra-antireflective property of the synthetic BCs at the UV and visible light range may suggest that their natural counterparts could have been optimized for antireflective and camouflage functions against leafhoppers’ predators (e.g., birds or insects), whose active vision spectra are also in the UV and visible light range^34^. To demonstrate the possible camouflage function of the BCs, we placed the synthetic BCs next to various leaf species (i.e., Chrysanthemum, Lantana, Callicarpa, and Fushia), and compared their colors through the simulated visions of a ladybird beetle (a predator of leafhopper)^35^. Note that the active vision spectrum of a ladybird beetle is from 311 to 605 nm^36^. Based on the images generated by the simulated vision of a ladybird, the appearance of the BCs and the green leaves have very high level of similarity both qualitatively (Fig. 5a) and quantitatively (Supplementary Fig. 23). To further support our hypothesis, we gathered the geometrical parameters of a number of representative natural integumental BCs, and found that their geometries (i.e., *R*_b_ and *R*_t_) are within the design parameters for strong omnidirectional antireflectance (<10%) of the synthetic BCs (Fig. 5b and Supplementary Fig. 24). This further suggests that the natural and synthetic BCs may possess similar antireflective properties, which could be important survival functions that protect leafhoppers or their eggs from being detected by their predators in their natural environments^18,23,24^.

Finally, the fabrication concept of synthetic BCs is very general and can be extended to other material systems compatible with electrochemical deposition technique (Fig. 5c). For example, BCs of nickel (Ni), gold (Au), manganese oxide (MnO_2_), and polypyrrole (PPy) can be prepared, where these materials have broad applications in energy and sensing applications^37,38^ (Supplementary Figs. 25–29). In addition, BCs of hybrid materials, for instance, Ag/MnO_2_ can be prepared by a step-by-step electrodeposition method (Supplementary Fig. 29). Translating the highly complex geometries of natural brochosomes into materials architectures of various compositions may lead to advanced applications in solar energy harvesting, imaging, and sensing devices^39–41^.

## Methods

### Preparation of DCC template

Glass slides (2.5 × 7.5 cm) were treated in a plasma cleaner (Harrick Plasma, PDC-32G) for 10 min to obtain a highly hydrophilic surface. Different amounts of aqueous dispersions composed of PS latex beads (2.5 wt%, purchased from Alfa Aesar) were spin-coated onto the treated glass slides (Supplementary Table 1). After dried in the ambient conditions, the glass slide covered by the PS spheres was immersed slowly into a water bath with the addition of ~0.01 g L^−1^ sodium dodecyl sulfate (SDS) at an angle of ~65° from the normal of the water surface. The PS spheres would assemble into a monolayer film at the air/water interface. A piece of silicon wafer was used to pick up the free-standing MCC template. Heat treatment at 110 °C for 3 min on a hot plate was performed to thermally anchor the PS spheres onto the silicon substrate before depositing a thin gold (Au) layer (usually 100 nm) onto the MCC template by a sputtering machine. Subsequently, the Au-coated MCC template was plasma-treated for 1 min to create a highly hydrophilic surface. Then, the Au-coated MCC template was used to pick up another layer of free-standing MCC template composed of smaller PS spheres floating at the air/water interface. During the drying process, the small PS spheres would assemble onto the large PS spheres to form a closely packed hexagonal array, leading to the formation of the DCC template.

### Preparation of the synthetic brochosomal coatings

The MCC template was prepared by a previously reported spin-coating method^25,26^. Then a thin layer of Au film was evaporated onto the MCC template, which was used as a seed layer to guide the subsequent electrodeposition process. Another layer of MCC template was transferred onto the Au-coated MCC template to form the DCC template. The DCC template was used as the cathode for metal electroplating. The electrolyte for silver plating contained 30 mM silver nitrate and 7 mM SDS. The electrolyte was maintained at a temperature of 45 °C on a hotplate. The deposition voltage and time was 1.5 V and 8 min unless otherwise specified. Electroplating of Au was performed in pure 10 mM gold(III) chloride (HAuCl_4_) solution at 2.5 V for 5 min. Nickel electrodeposition was carried out in 1.1 M nickel(II) sulfate (NiSO_4_), 10 mM SDS, and 0.65 M boric acid (H_3_BO_3_) with the pH value adjusted to 3 using sulfuric acid (H_2_SO_4_) at 3 V for 15 min. The DCC template was used as the anode for PPy and metal oxide deposition. For PPy deposition, the electrolyte was formed by 0.1 M pyrrole and 0.1 M sodium dodecylbenzene sulfonate. The deposition voltage for PPy was 1.5 V for 1 min. Electroplating of MnO_2_ was conducted in 0.1 M manganese acetate at 3 V for 4 min. After immersing the electroplated DCC template into dichloromethane for 1 min to remove the PS spheres, BCs of the electroplated material are obtained. Step-by-step electroplating can create BCs of hybrid materials. For example, Ag/MnO_2_ BCs were created by electroplating of Ag for 5 min and MnO_2_ for 3 min using the above electrolytes after dissolving the DCC template using dichloromethane.

### Preparation of control samples

The Ag mesoporous films were prepared using MCC template on a piece of Au-coated silicon wafer as the cathode electrode with the same electrolyte for the growth of Ag BCs. The deposition voltage was 1.5 V and the electroplating time was 3.5 min. The electrodeposited Ag nanoporous film was deposited on a piece of Au-coated silicon wafer in the same electrolyte solution at 1.5 V for 10 min. The 100-nm thick Ag mirror was thermally evaporated on a piece of silicon wafer.

### Fabrication of black silicon

Black silicon was prepared using a deep reactive ion etching method, according to a previous publication^42^.

### UV–Vis–NIR measurements

The UV–Vis–NIR reflection spectra in the wavelength range of 250–2000 nm were measured (Lambda 950, Perkin-Elmer). The incident angles can be varied from 8° to 68°.

### FDTD simulations

FDTD simulations were carried out using the commercial software Lumerical Solutions. Constrained by the computer resources, we did not consider the roughened sidewall structure of the brochosome, which consisted of numerous Ag nanoparticles ~30 to ~70 nm in size. Instead, we built the FDTD model of BCs using smooth Ag shells with ordered pits for simplicity (Supplementary Fig. 15). As a result, our model did not capture the antireflection contributions due to the roughened sidewall of the BCs in the experiments, which is known to effectively suppress reflection^29^. While the use of smooth Ag shells as FDTD models gives rise to significantly increased reflection compared to the experimental measurements, the qualitative trend of the reflection spectra can still be revealed by the FDTD simulation results. The ordered pits were created using the open source software Blender. In this model, we first created an MCC template composed of small spheres in the software. Then, a small force was applied to place the MCC template onto the large PS spheres and simultaneously apply a weak attraction force between the small PS spheres to maintain a close packing of the MCC template. We then increased the diameter of the large PS spheres to the extent that it can bury half of the small PS spheres. Finally, we converted the large PS spheres into Ag shells with a shell thickness equal to the radius of the small PS spheres. We set the materials property of the small PS to be vacuum to better represent the actual BC configurations in our experiments. The models were then input to the software for FDTD simulations.

### UV photography

To mimic the vision of insects and birds, we first modified the camera (Canon T4i) to be UV sensitive by LifePixel (Mukilteo, WA). Then we used a software micaToolbox developed by Troscianko and Stevens to simulate the vision as observed by insects and birds^35^. To create the simulated images, we first used the UV and regular camera to take photographs on the Ag BCs lying on a green leaf under sunlight, respectively. Then the toolbox converted the photos to multispectral objective images consisting of all RGB and UV channels. Finally, the false-color-simulated images were produced based on the specific spectral sensitivities of the visual system of a given insect, as shown in Fig. 5 and Supplementary Fig. 23.

### Evaluation of color difference in non-human visual systems

We used a published method for comparing colors in non-human visual systems, which is based on Vorobyev and Osorio’s receptor noise model^34,43^ implemented in MICA. This method calculates the distance between two colors with the unit JND (just noticeable difference). This unit is based on the signal-to-noise ratios of each channel of the specific animal visual system. If the JND value between two colors is less than 1, then the images are said to be indistinguishable. However, if the value is above ~3, then the images are said to be discriminable under good lighting conditions^44^. The Weber fraction (i.e., the ratio of intensity between two lights perceived by a visual system at threshold)^44^ used is 0.05 based on ladybird beetle and human’s visual system^36,45^. Data are measured from 32-bit normalized objective images processed by MICA Toolbox.

### Data availability

The data that support the findings of this study are available from the corresponding authors upon reasonable request.

## Electronic supplementary material

Supplementary Information
