# Supplementary material for: Ultra-antireflective synthetic brochosomes
Source: Nat Commun. 2017 Nov 3;8:1285. doi: 10.1038/s41467-017-01404-8 (PMC5670136; doi:10.1038/s41467-017-01404-8)
Supplement: Supplementary file 1 — Supplementary Information [file 41467_2017_1404_MOESM1_ESM.pdf]

Supplementary Note 1: Structural control of BCs. The availability of PS spheres in various sizes (from < 100 nm to > 10 μm) allows us to design synthetic BCs with a broad range of structural geometries. For instance, Ag brochosomes composed of pits ~ 290 nm in size can be prepared by using 350 nm PS spheres (Supplementary Fig. 2), resulting in > 30 pits that are regularly arranged on a single Ag brochosome particle. The pit size can be further reduced to ~ 180 nm by using 200 nm PS spheres (Supplementary Fig. 3). In this case, the pit number will be > 50. In addition, we have demonstrated the formation of BCs using PS beads with diameters ranging from 1 μm to 4.5 μm with various pit sizes (Supplementary Figs 8 – 10).

Supplementary Note 2: Structural influence on the omnidirectionality of the antireflection performance. Experimentally, increasing the pit depth was found to enhance the antireflection performance. In particular, optimized antireflection properties can be achieved when the ratio of the pit depth/ pit diameter is ~ 0.55 (Supplementary Fig. 12). To characterize the effect of the pit number on the antireflection performance of the BCs, we chose to increase the diameter of the brochosome particle while keeping the pit size constant. Since the pit size ($2R_{\mathrm{t}}$) and pit number (n) can be related by $n = \frac{\pi}{2\sqrt{3}}(\frac{R_{\mathrm{b}}}{R_{\mathrm{t}}})^2$ for a given size ($2R_{\mathrm{b}}$) of brochosome particle, increasing the particle size increases the pit number correspondingly. Specifically, we compared the antireflection performance of BCs formed by particle sizes of 2 μm and 4.5 μm (with 200 nm pit size), respectively, and found that the antireflection performance in the range of 250 nm < λ < 2000 nm (λ is wavelength of the electromagnetic waves) has been improved for the 4.5 μm sized particle due to higher pit numbers. Therefore, increasing the pit number is favorable to suppress the reflection, which is also shown in the FDTD simulation result (Supplementary Figs 15 – 18). Since the pit size, pit number, and the diameter of the brochosomes are interrelated, there is an optimal parameter to suppress the antireflection corresponding to a specific wavelength range. In

the wavelengths of our interest (i.e., 250 nm < λ < 2000 nm), the best antireflection performance of BCs is comprised of 2 µm brochosomes with pits of 470 nm in size and 260 nm in depth.

Supplementary Table 1. Total volume of 2.5 wt% PS beads solution required to form the MCC template on the glass slides through spin-coating.

**Diameter** (nm)	200	350	500	1000	2000	4500
**Volume** (μl)	20	20	25	30	40	80

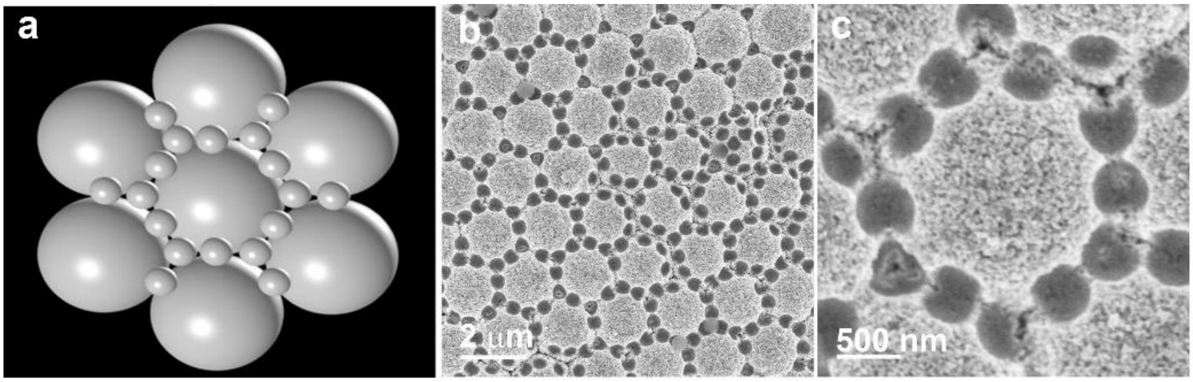

Supplementary Fig. 1. Silver (Ag) surface patterns obtained by electrodeposition using conventional binary colloidal crystal templates. a. Schematic of the binary colloidal crystal template. **b**, **c**, Scanning electron micrographs of the Ag surface pattern at different magnifications. The PS template has been removed.

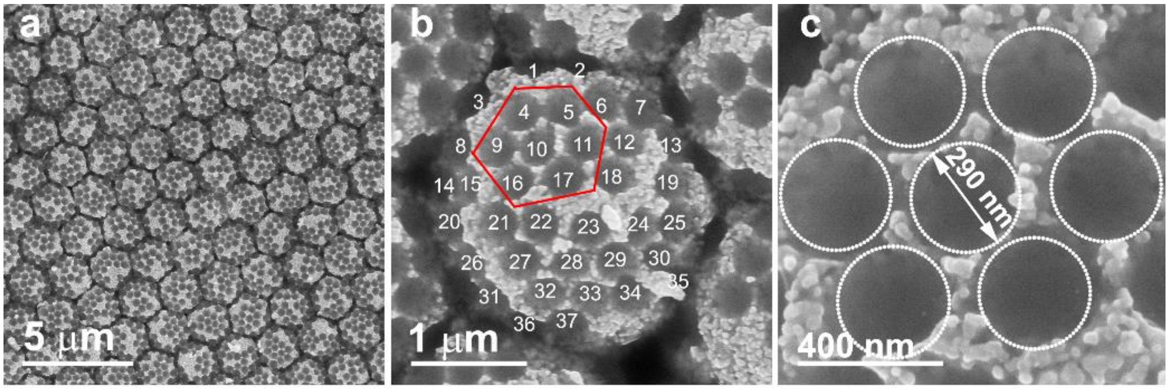

Supplementary Fig. 2. BCs obtained using the DCC template with the top MCC template formed by 350 nm PS spheres and the bottom MCC template formed by 2 μm spheres (named as 350 nm/2 μm DCC template). a-c, Scanning electron micrographs of the BCs at increasing magnifications.

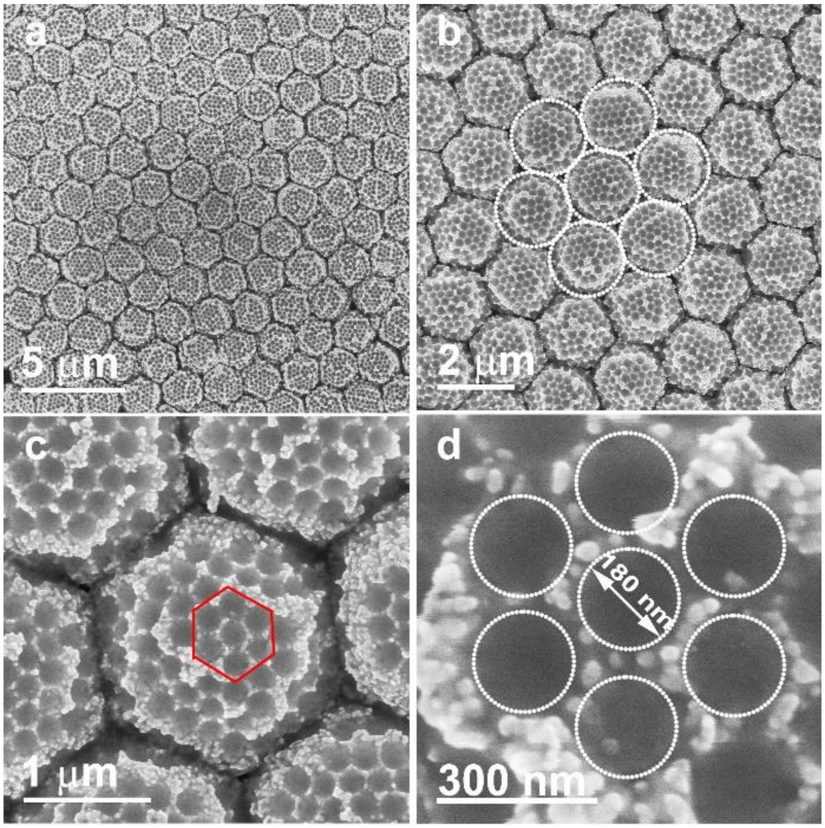

Supplementary Fig. 3. BCs obtained using the DCC template with the top MCC template formed by 200 nm PS spheres and the bottom MCC template formed by 2 μm spheres (named as 200 nm/2 μm DCC template). a-d, Scanning electron micrographs of the BCs at increasing magnifications.

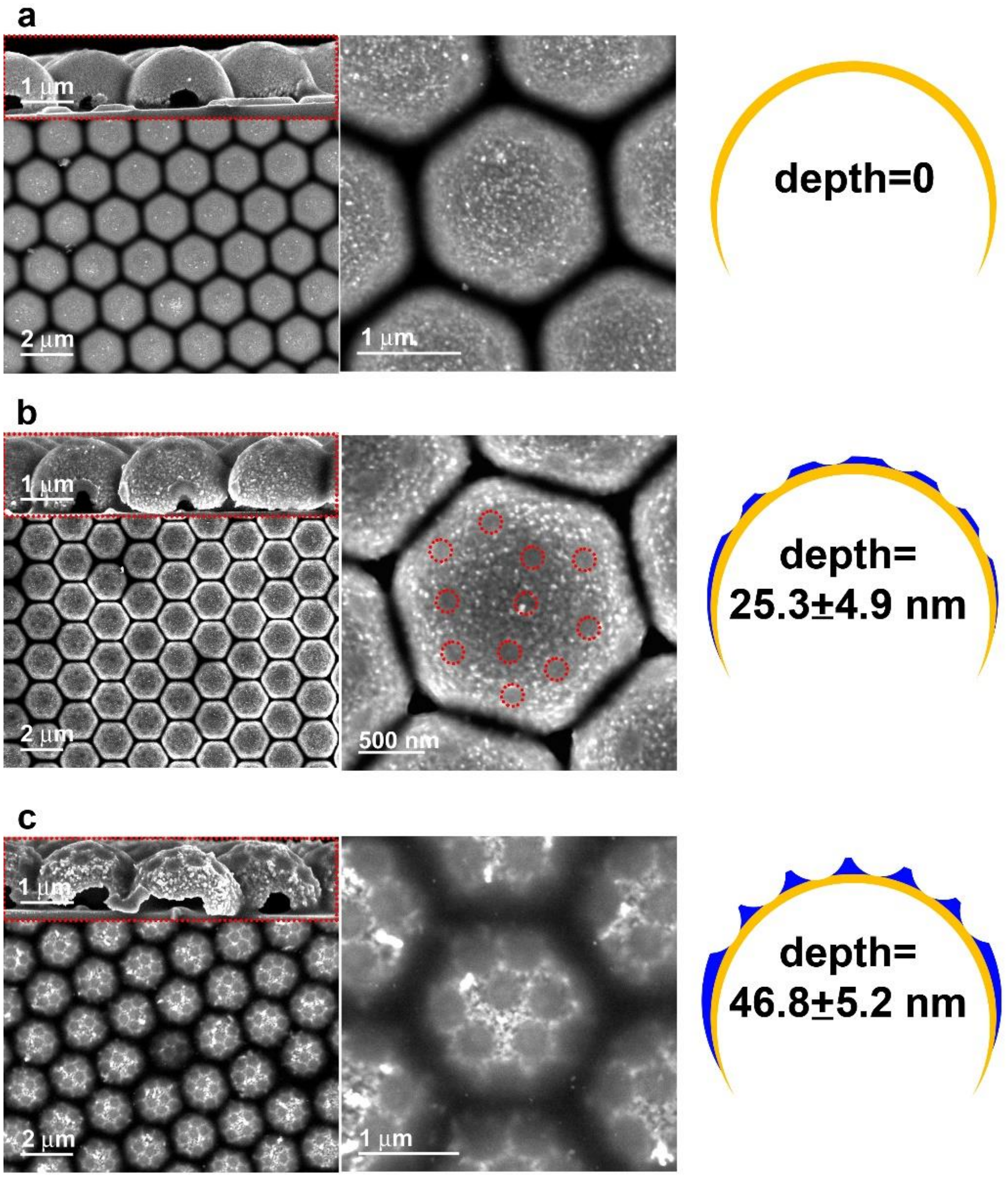

Supplementary Fig. 4. Scanning electron micrographs and schematics showing the evolution of the pit depth of BCs obtained during electrodeposition at 1.5 V at different deposition times. a, 0 min. **b**, 2 min. **c**, 4 min.

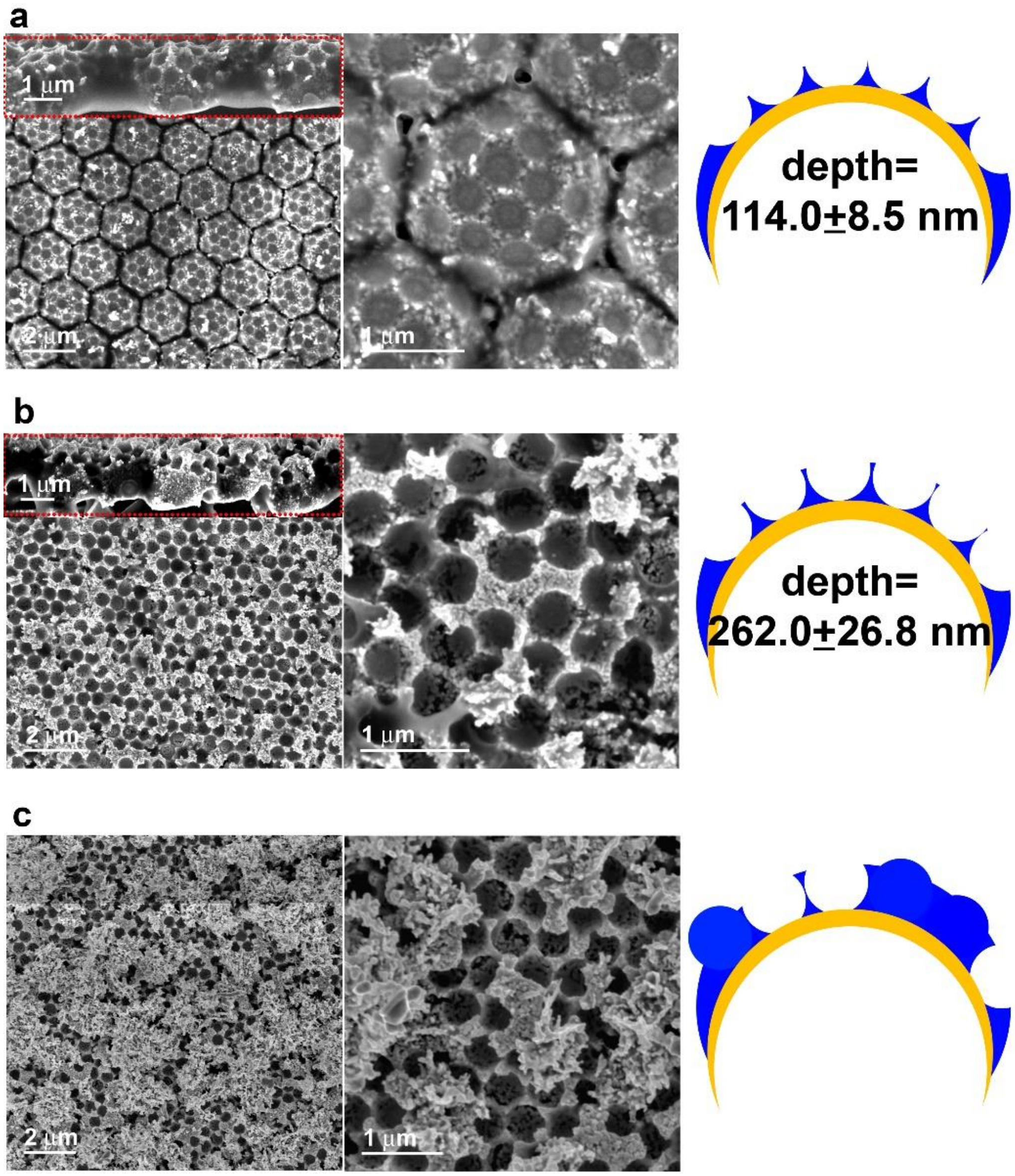

Supplementary Fig. 5. Scanning electron micrographs and schematics showing the evolution of the pit depth of BCs obtained during electrodeposition at 1.5 V at different deposition times. a, 4 min. **b**, 8 min. **c**, 10 min.

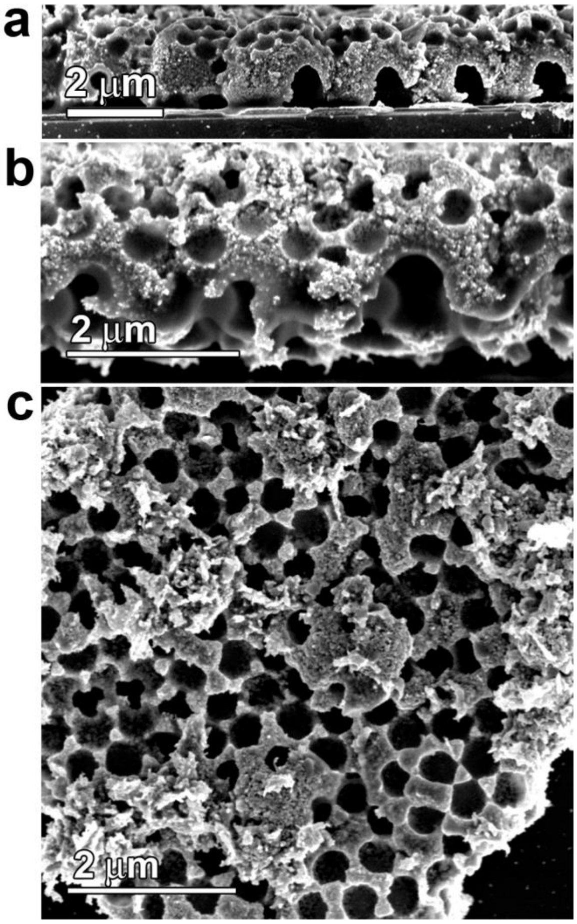

Supplementary Fig. 6. Scanning electron micrographs showing the pit depth of BCs obtained during electrodeposition at 2 V for different times. a, 4 min (side view); **b**, 6 min (side view); **c**, 6 min (top view).

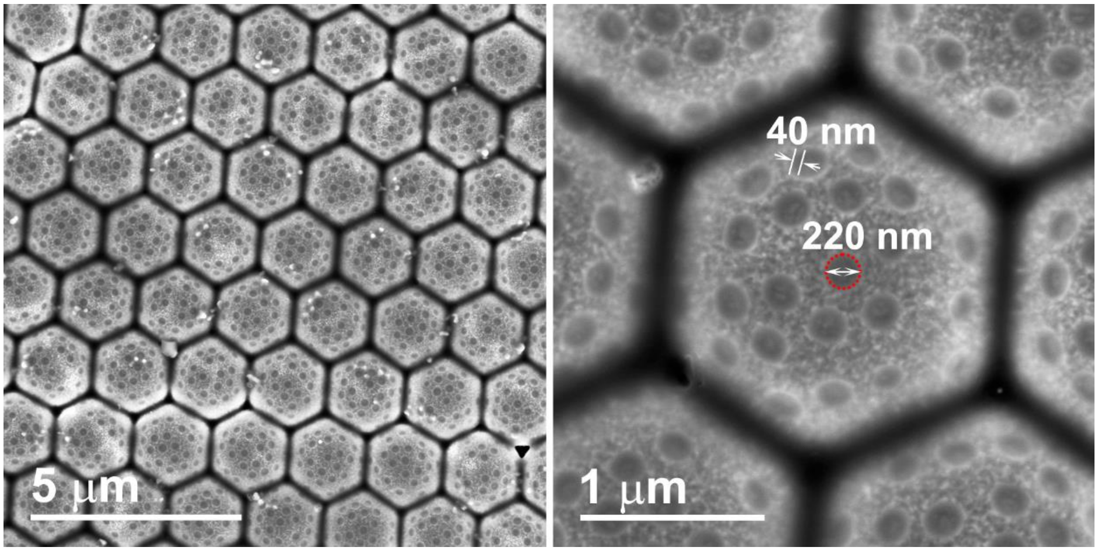

Supplementary Fig. 7. Scanning electron micrographs showing the BCs obtained during electrodeposition at 1 V for 10 min.

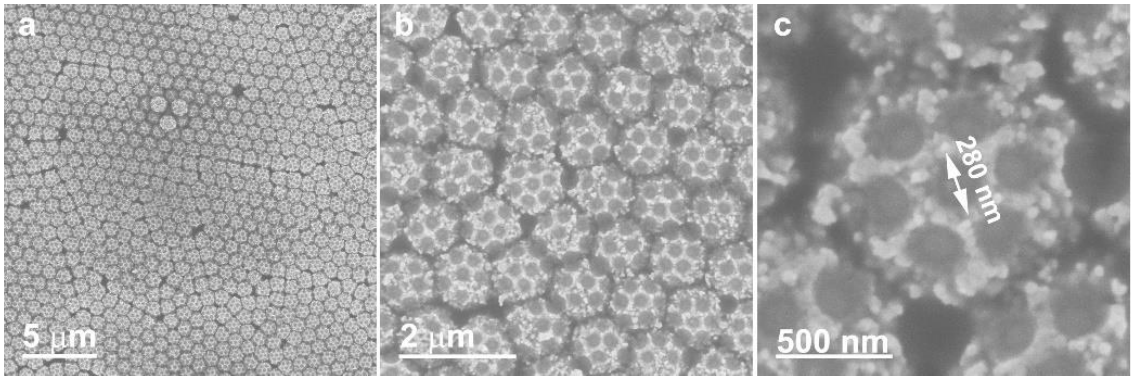

Supplementary Fig. 8. BCs obtained using the DCC template with the top MCC template formed by 350 nm PS spheres and the bottom MCC template formed by 1 μm spheres (referred to as 350 nm/1 μm DCC template). a-c, Scanning electron micrographs of the BCs at increasing magnifications..

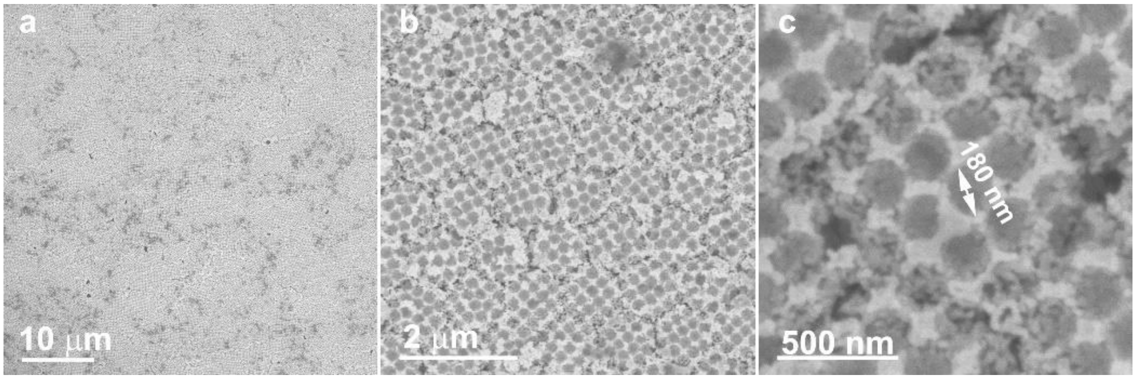

Supplementary Fig. 9. BCs obtained using the DCC template with the top MCC template formed by 200 nm PS spheres and the bottom MCC template by 1 μm spheres (referred to as 200 nm/1 μm DCC template). a-c, Scanning electron micrographs of the BCs at increasing magnifications.

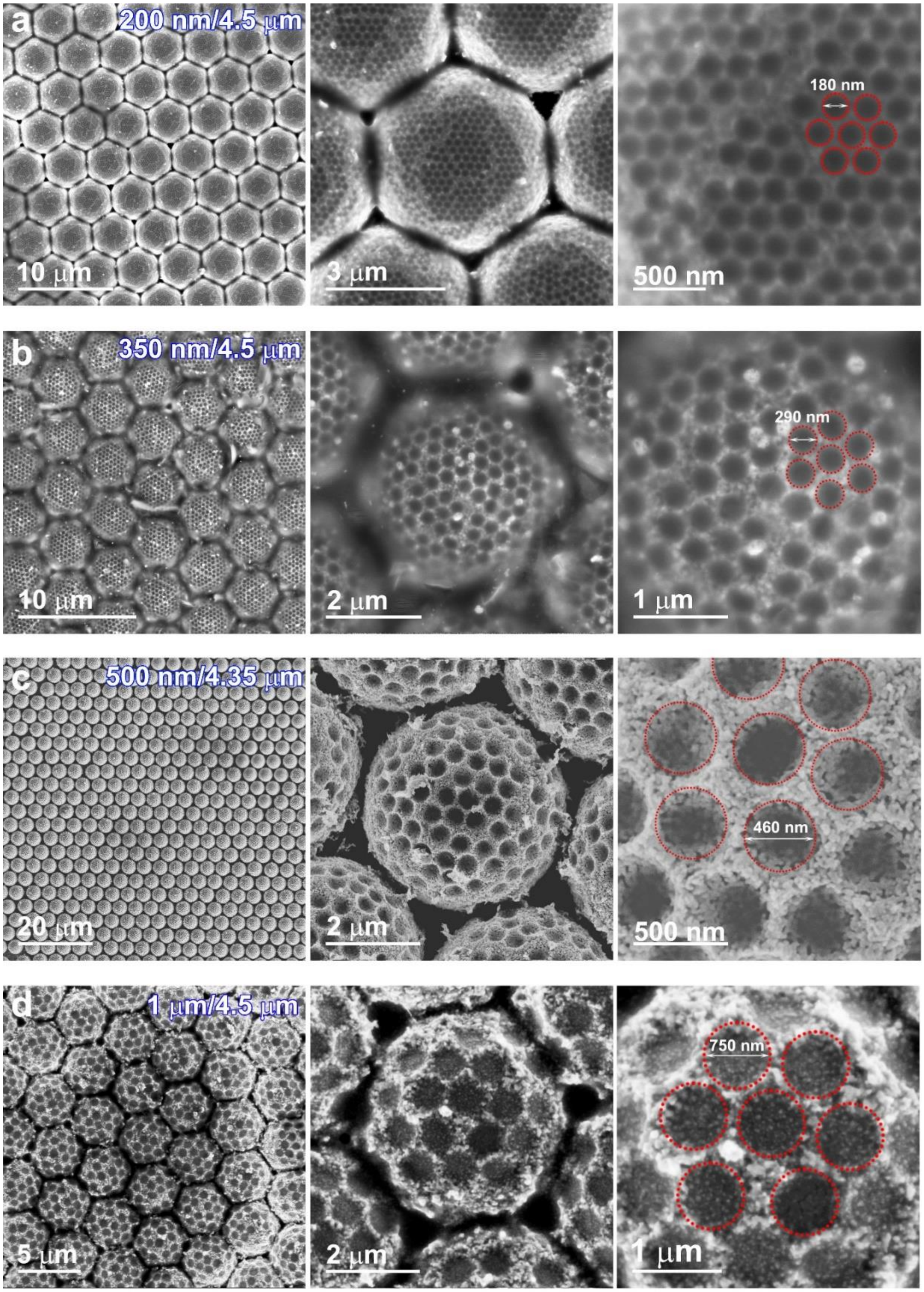

Supplementary Fig. 10. BCs formed by 4.5 μm brochosomes with pits formed using different size PS spheres: **a**, 200 nm. **b**, 350 nm. **c**, 500 nm, and **d**, 1 μm. Note that the 4.5 μm PS spheres are plasma-etched for 10 min to create non-close-packed brochosomes.

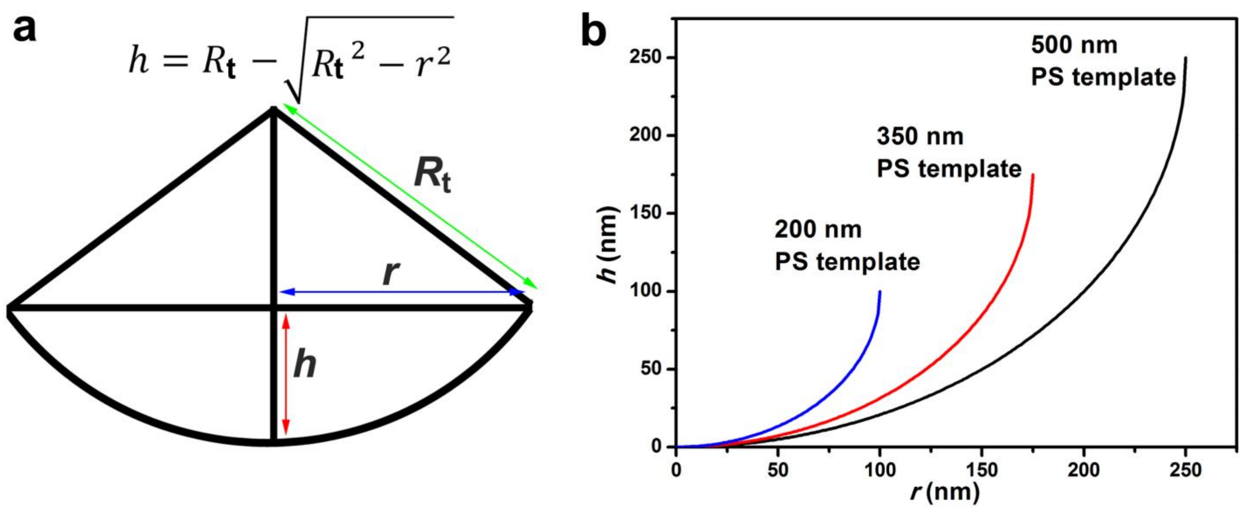

Supplementary Fig. 11. Predicting the pit depth by the opening size of the pit. a, Schematic showing the geometrical relationship of various parameters of the pit. **b**, Theoretical prediction showing the relationship between the pit depth and the opening size of the pits created by 200 nm, 350 nm, and 500 nm PS spheres.

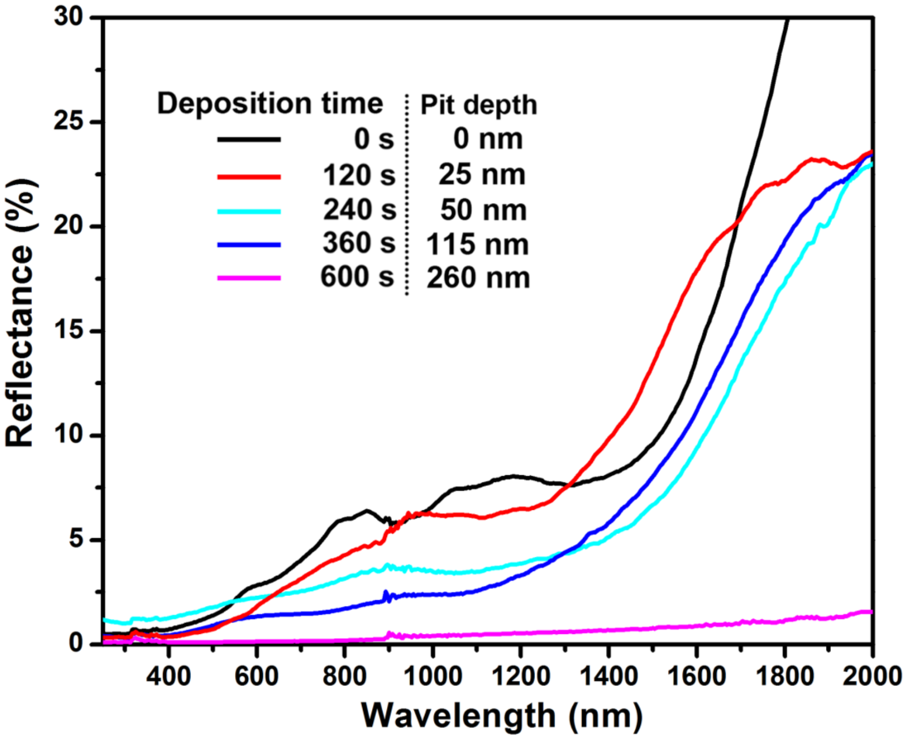

Supplementary Fig. 12. Influence of pit depth on the reflectance spectra of BCs. The brochosome diameter is 2 µm with pit size of 500 nm at various depths as indicated in the figure.

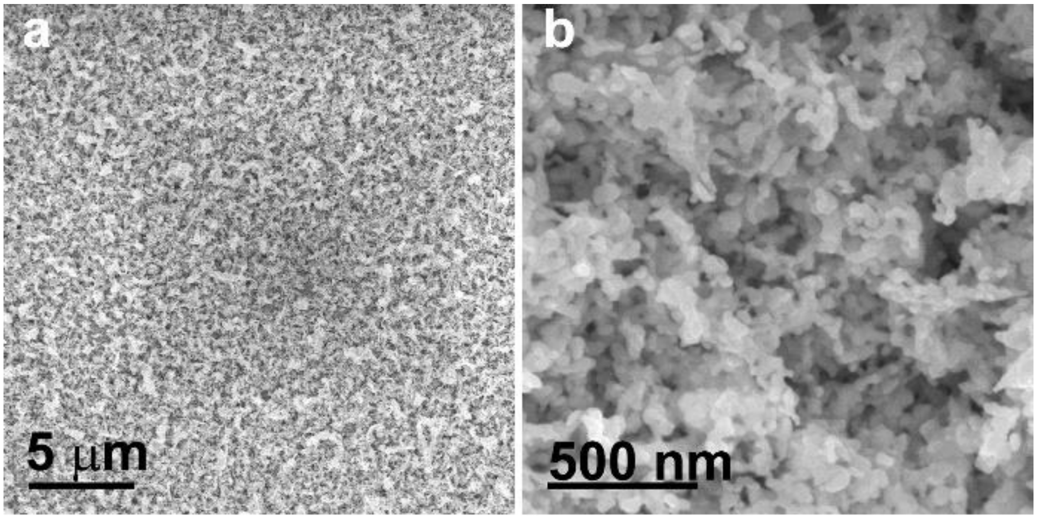

Supplementary Fig. 13. SEM image of the Ag nanoporous film obtained by electrodeposition of Ag onto the gold-coated silicon substrate. a, **b**, Scanning electron micrographs of the BCs at increasing magnifications.

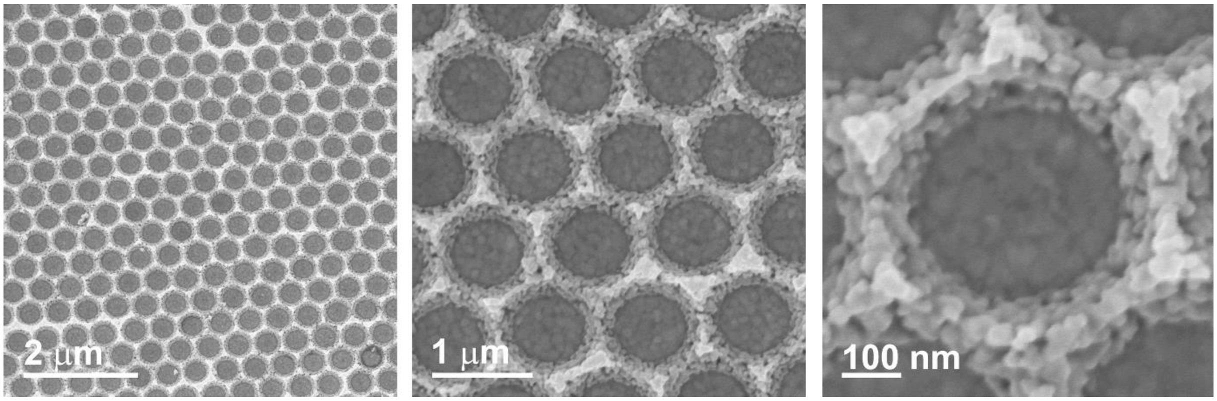

Supplementary Fig. 14. Ag honeycomb mesoporous film obtained by electrodeposition using the MCC template on the Au-coated silicon substrate shown at different magnifications. Note that the PS spheres have been removed from the MCC template to form the nanoscale pits. The pit depth of the mesoporous film is ~ 250 nm.

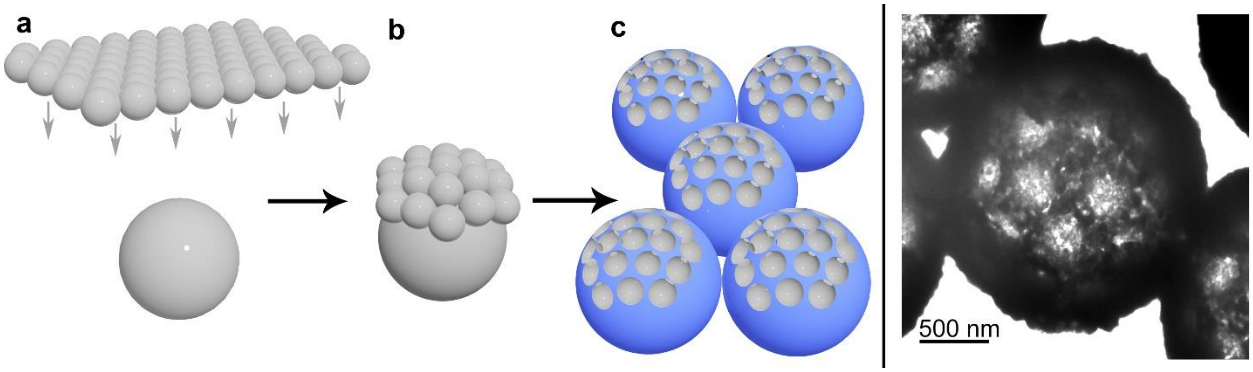

Supplementary Fig. 15. Model generation of the BCs for FDTD simulations. a, MCC template is placed on a large PS sphere by exerting a force in the Blender software. **b**, Small PS spheres are hexagonally packed on the large PS sphere. **c**, The BCs are created by altering the large PS sphere into a thin Ag shell and the small PS spheres are set to be vacuum to create the ordered pits. The transmission electron microscope image of an Ag brochosome particle is shown at the right hand side.

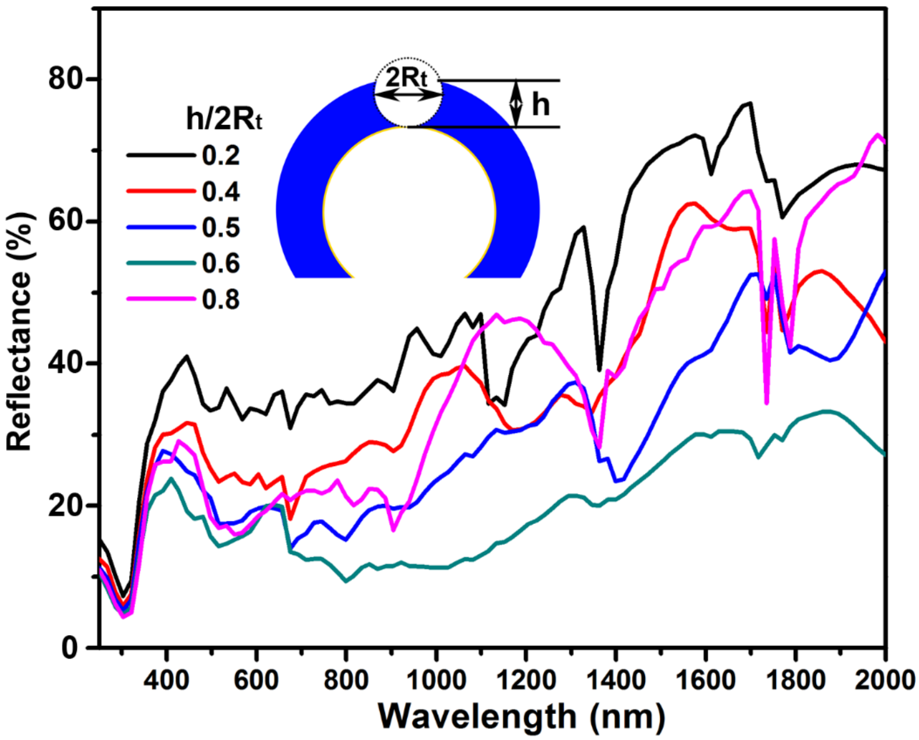

Supplementary Fig. 16. FDTD simulation showing the influence of pit depth on the antireflection performance of the BCs. The diameter of the brochosome is 2 μm with pit size of 500 nm at different pit depths.

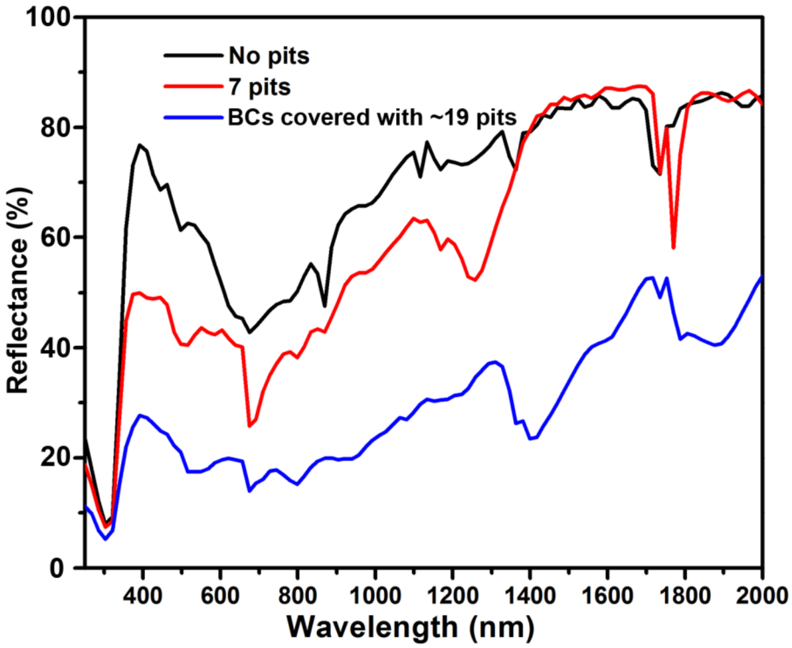

Supplementary Fig. 17. FDTD simulation showing the influence of pit number on the antireflection performance of the BCs. The diameter of the brochosome is 2 µm with pits formed using various amounts of 500 nm PS spheres.

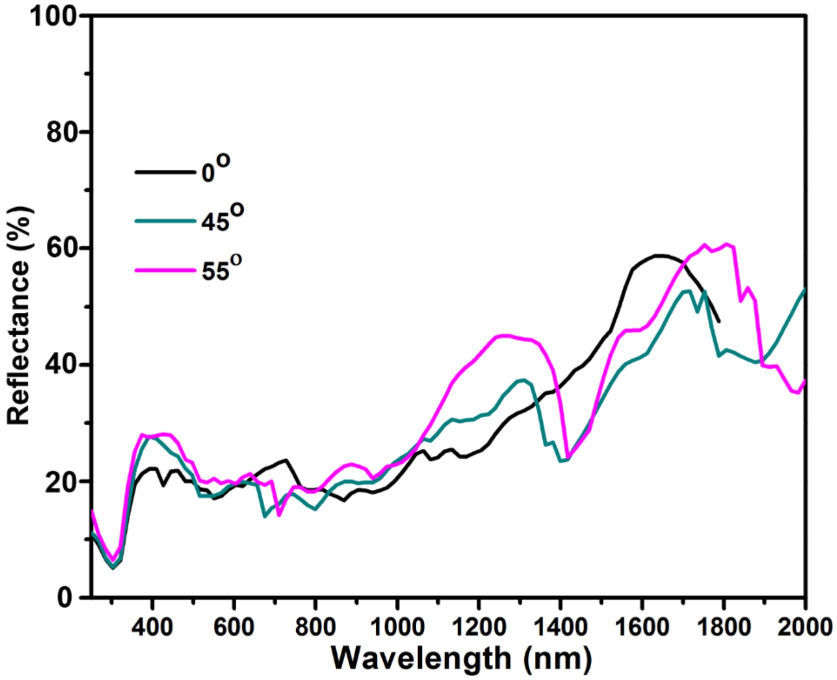

Supplementary Fig. 18. FDTD simulation showing the angle dependence of the reflection spectrum of BCs. The diameter of the brochosome is 2 µm and is fully covered with pits of 500 nm in size.

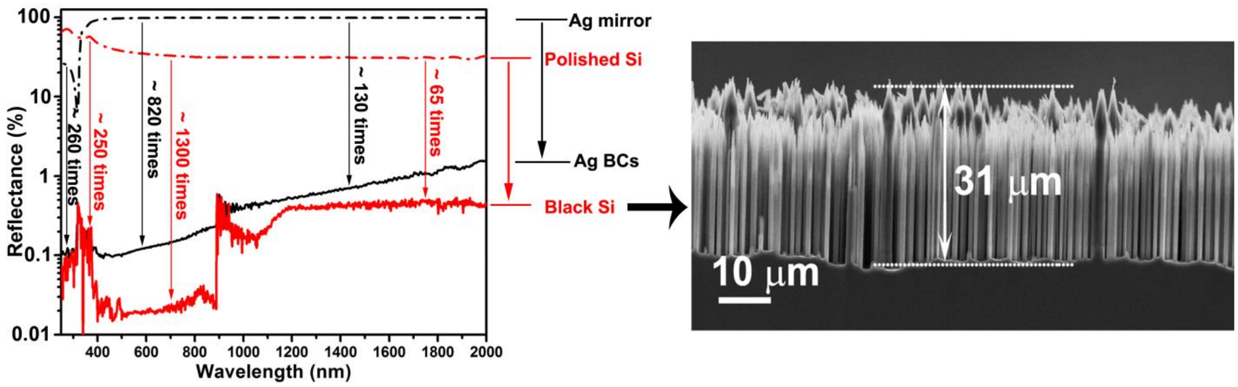

Supplementary Fig. 19. Comparison of the specular reflection spectra of the Ag BCs, Ag mirror, the black Si, and polished silicon. The cross-sectional scanning electron micrograph of the black silicon is shown on the right hand side. We showed that the reflection reduction from flat silver to silver brochosomes are comparable to those of from flat silicon to silicon nanowires in the wavelength range of ~350 nm to 2000 nm. Here, we define the reflection reduction of silver substrates, $A_{silver,}$ as the ratio of flat silver reflectance to silver brochosomes reflectance; $A_{silicon}$ refers to the ratio of flat silicon reflectance to silicon nanowires reflectance. Specifically, the synthetic brochosomes showed better reflection reduction performance than the silicon nanowire in the ultraviolet and near infrared range (i.e., A_{silver} ~ 260 and $A_{silicon}$ ~ 250 in the UV range; and A_{silver} ~ 130 and $A_{silicon}$ ~ 65 in the near IR range). In the visible light range, the silicon nanowires have better reflection reduction performance (i.e., A_{silver} ~ 820 and $A_{silicon}$ ~ 1300). These ratios were determined approximately by averaging the reflectance in the UV, visible, and near IR range, respectively. One of the key advantages of our fabrication approach is the ability to use broader range of materials to create antireflective coatings.

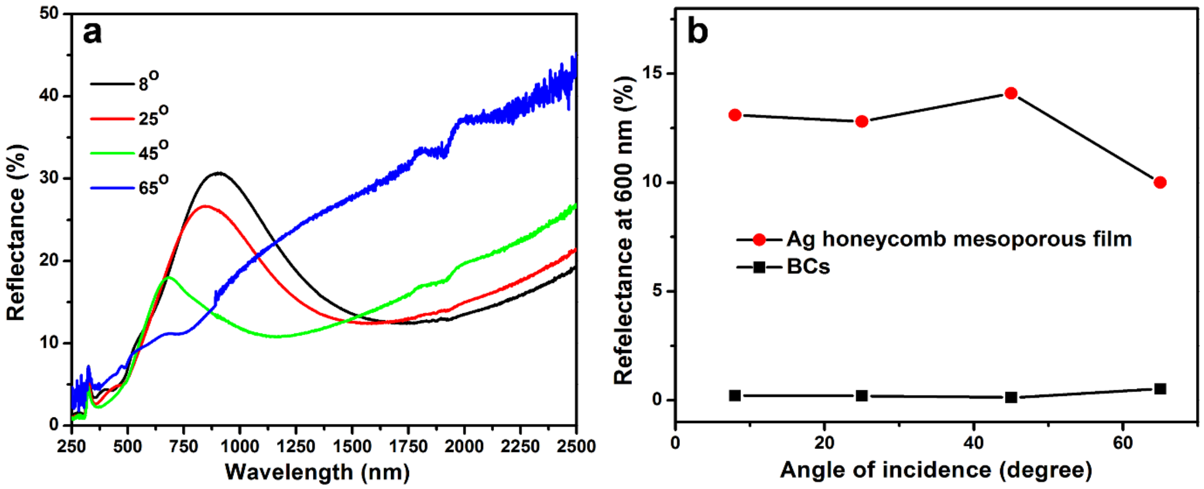

Supplementary Fig. 20. Comparison of the angle dependence of the reflection spectra of BCs and the Ag mesoporous film (the surface morphology is shown in Fig. S14). a, Reflection spectra of the Ag honeycomb mesoporous film at different incident angles. **b**, Reflectance of the BCs and Ag mesoporous film at 600 nm at different incident angles.

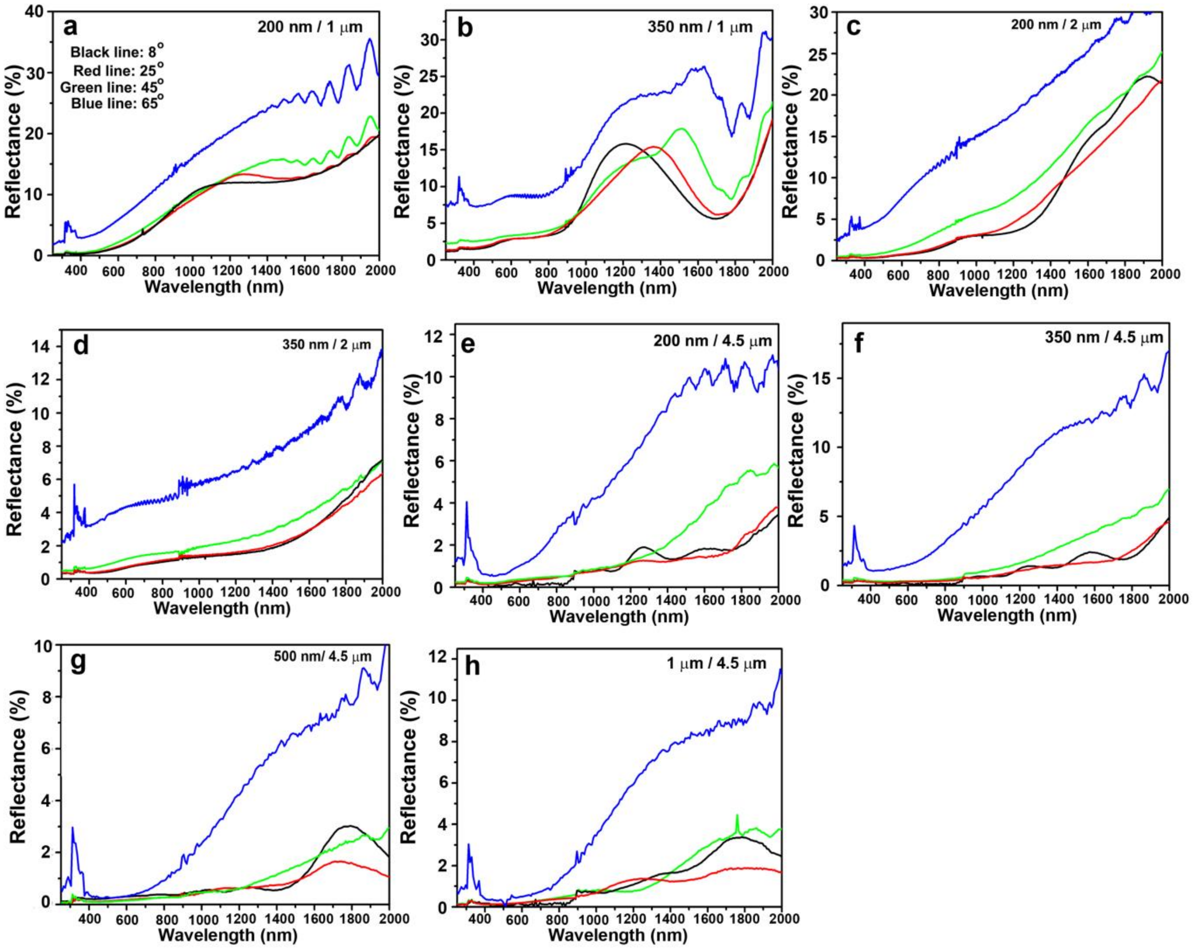

Supplementary Fig. 21. Angle dependence of the reflectance spectra for brochosomes with different diameters decorated with different sized pits. a, **b**, Reflectance spectra of 1 μm brochosomes with pit size of 200 nm and 350 nm, respectively. **c**, **d**, Reflectance spectra of 2 μm brochosomes with pit size of 200 nm and 350 nm, respectively. **e-h**, Reflectance spectra of 4.5 μm brochosomes with pit size of 200 nm, 350 nm, 500 nm, and 1 μm respectively.

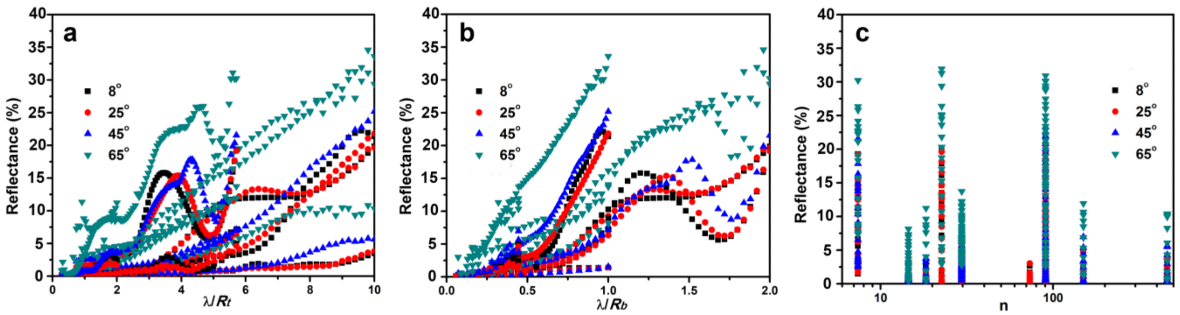

Supplementary Fig. 22. Parametric study of the relationship between the geometrical parameters of brochosomes and the optical reflection. a, the reflectance-λ/R_t relationship. **b**, the reflectance- λ/R_b relationship. **c**, the reflectance-n relationship.

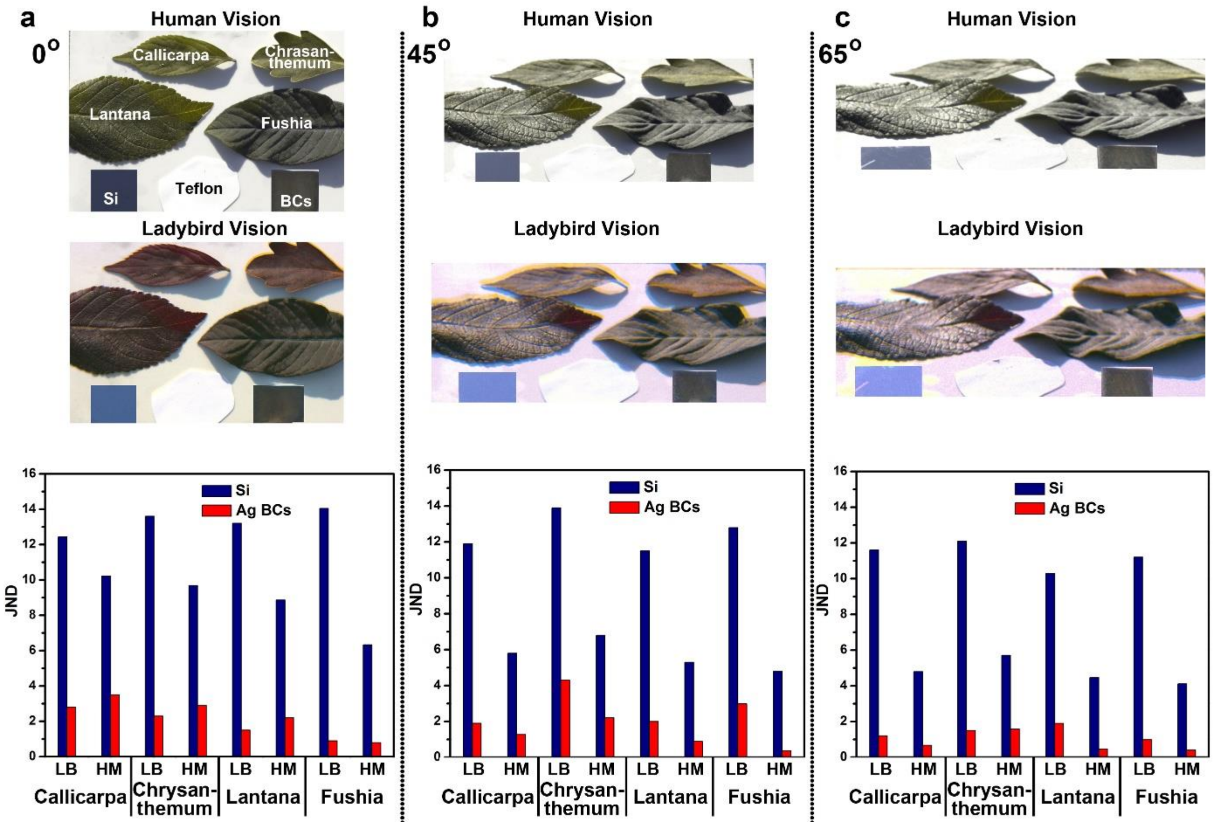

Supplementary Fig. 23. Quantitative evaluation of the colour difference between BCs and different leaf species at different incident angles in the vision of human and ladybird beetle. **a,** 0°. **b,** 45°. **c,** 65°. LB: ladybird vision. HM: human vision.

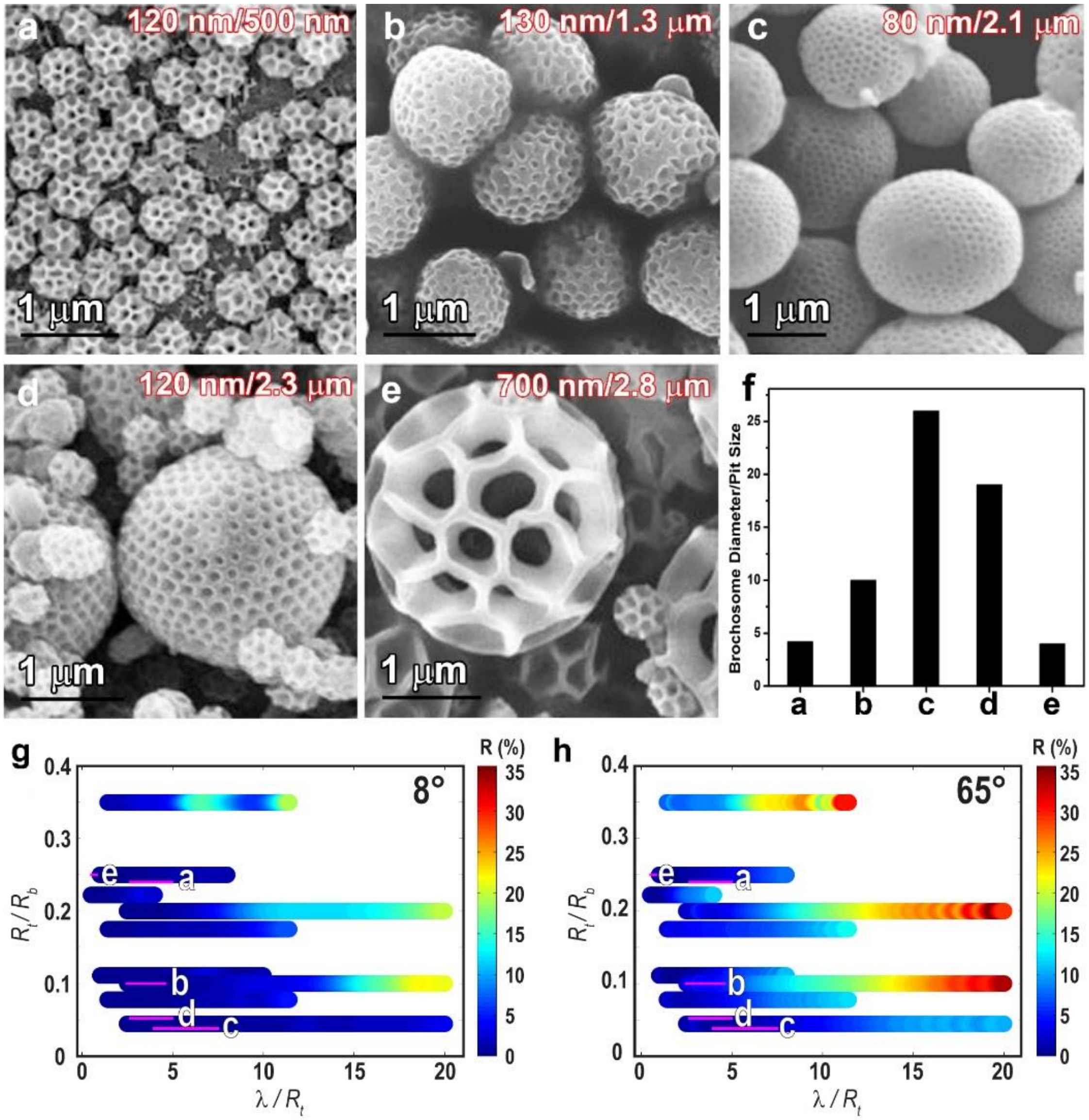

Supplementary Fig. 24. Geometrical parameters of natural brochosomes are within the strong antireflection region in the reflection-structure colour map created based on the experimental results. a-e, brochosomes secreted by different leafhoppers. **a**, *Oncometopic orbona*, male. **b**, *Proconia esmeraldae*, female. **c**, *Homalodisca coagulata*, male. **d**, *Proconia esmeraldae*, male. **e**, *Diestostemma stesilea*, male. **f**, Summary of the ratio between the diameter and the pit size of the brochosomes. Image reprinted from R.A. Rakitov, Powdering of egg nests with brochosomes and related sexual dimorphism in leafhoppers (Hemiptera: Cicadellidae), *Zool. J. Linn. Soc.* **140**, 353-381 (2004), by permission of the Linnean Society. **g** and **h,** location of the geometrical parameters of different leafhoppers on the reflection-structure map at different incident angles; the magenta line represents the natural brochosome geometry (x-axis) and the λ/R_t range over which they are visible to ladybirds (a leafhopper predator), i.e., 311 nm/R_t to 605 nm/R_t, where R_t depends on the natural brochosome species (letters **a-e** refer to species in panels **a-e**, respectively). **g**, 8°. **h**, 65°.

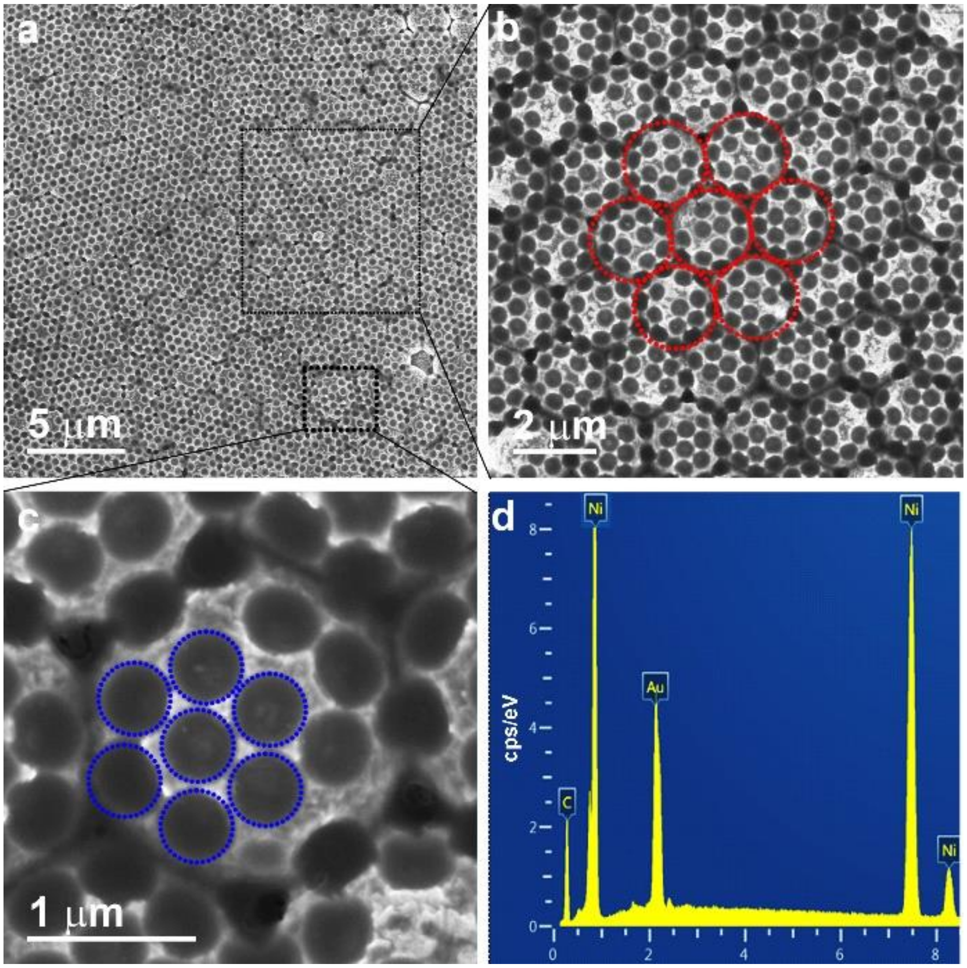

Supplementary Fig. 25. Electrodeposited nickel BCs. a-c, Scanning electron micrographs of the BCs at increasing magnifications. **d**, EDX spectrum of the electrodeposited nickel BCs.

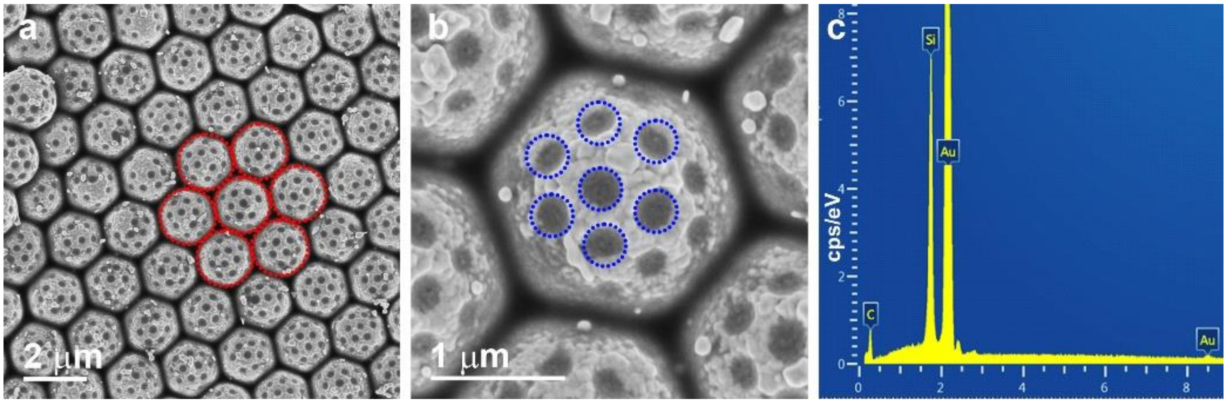

Supplementary Fig. 26. Electrodeposited gold BCs. a, **b**, Scanning electron micrographs of the BCs at increasing magnifications. **c**, EDX spectrum of the electrodeposited gold BCs.

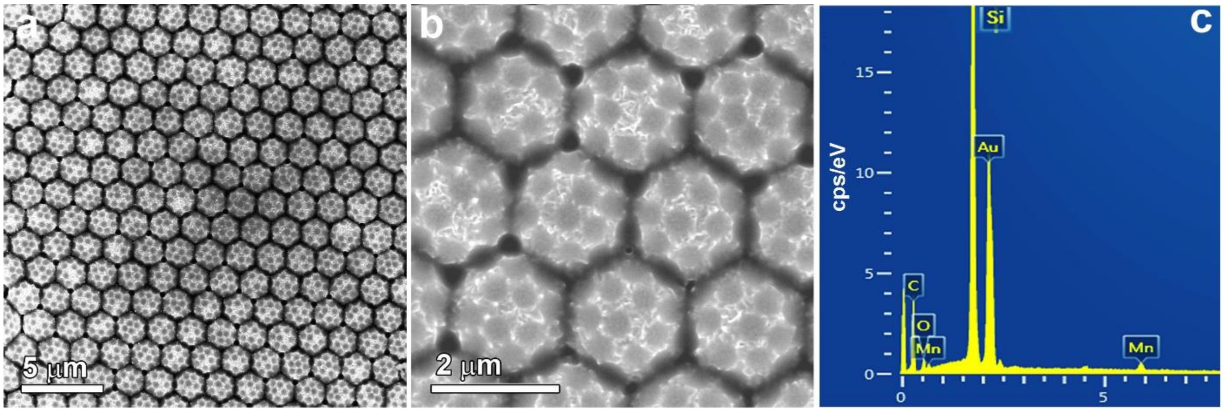

Supplementary Fig. 27. Electrodeposited manganese oxide BCs. a, **b**, Scanning electron micrographs of the BCs at increasing magnifications. **c**, The corresponding EDX spectrum.

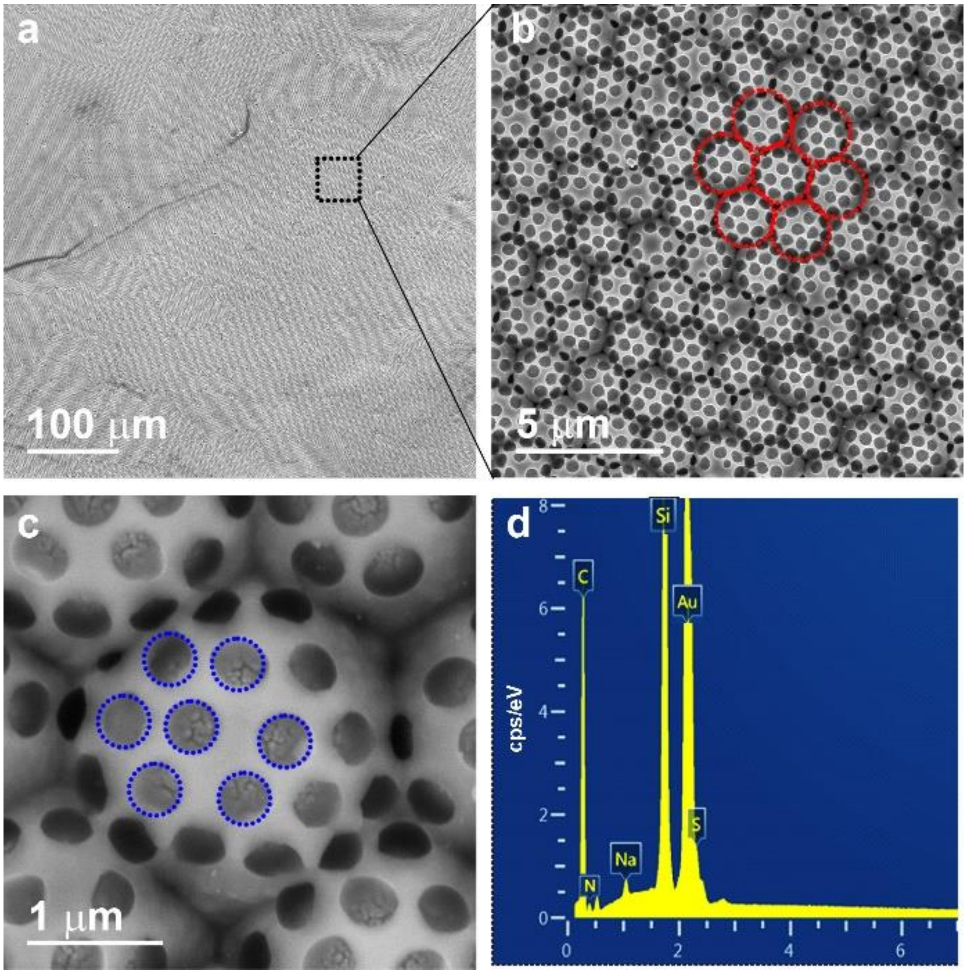

Supplementary Fig. 28. Electrodeposited polypyrrole BCs. a-c, Scanning electron micrographs of the BCs at increasing magnifications. **d**, The corresponding EDX spectrum.

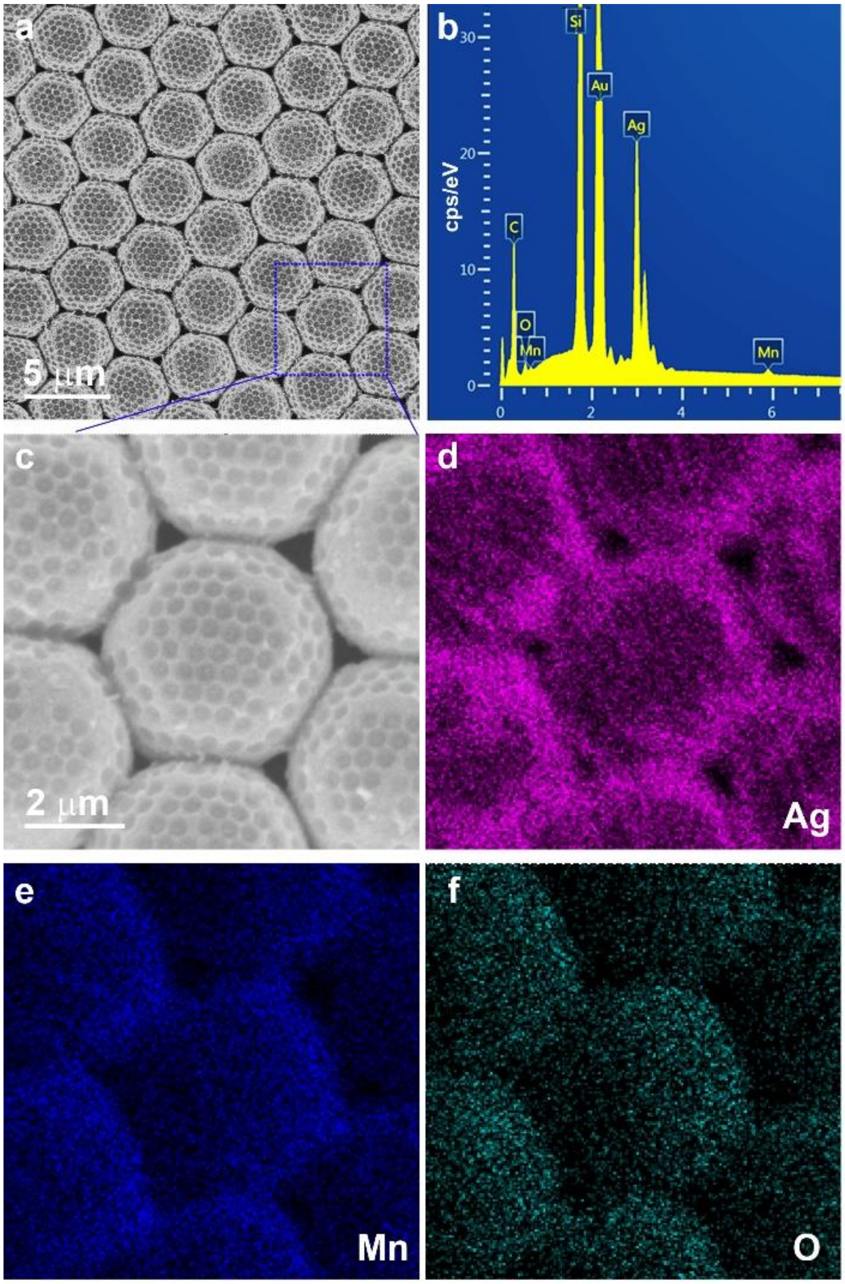

Supplementary Fig. 29. Hybrid BCs of Ag/MnO_2. **a**, SEM image. **b**, EDX spectrum. **c**, Magnified SEM image. **d-f**, Element mapping results indicating that the BCs consists of multiple elements.
